# Sensitive Measurement of Drug-Target Engagement by a Cellular Thermal Shift Assay with Multiplex Proximity Extension Readout

**DOI:** 10.1021/acs.analchem.1c02225

**Published:** 2021-07-28

**Authors:** Rasel A. Al-Amin, Caroline J. Gallant, Phathutshedzo M. Muthelo, Ulf Landegren

**Affiliations:** Department of Immunology, Genetics and Pathology, Science for Life Laboratory, Uppsala University, Uppsala SE-751 08, Sweden

## Abstract

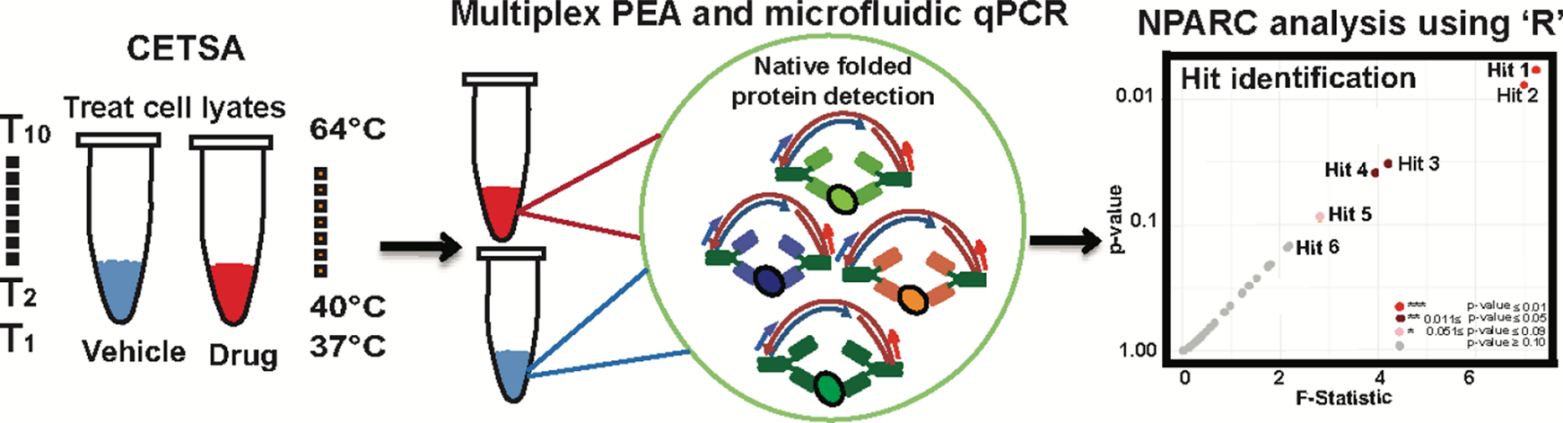

The ability to monitor target engagement in cellular contexts is a key for successful drug discovery and also valuable in clinical routine. A cellular thermal shift assay (CETSA) provides realistic information about drug binding in cells and tissues, revealing drug-target engagement in clinically relevant samples. The CETSA combined with mass spectrometry (MS) detection can be applied in the early hit identification phase to generate target engagement data for large sets of proteins. However, the analysis is slow, requires substantial amounts of the sample material, and often misses proteins of specific interest. Here, we combined the CETSA and the multiplex proximity extension assay (PEA) for analysis of target engagement of a set of 67 proteins from small amounts of the sample material treated with kinase inhibitors. The results were concordant with the corresponding analyses read out via MS. Our approach allows analyses of large numbers of specific target proteins at high sensitivity in limited sample aliquots. Highly sensitive multiplex CETSA-PEA assays are therefore promising for monitoring drug-target engagement in small sample aliquots in the course of drug development and potentially in clinical settings.

## Introduction

Drug development programs often fail for reasons of safety or efficacy during clinical phases I–III, and half of these failures have been reported to be due to lack of efficacy.^1,2^ The efficacy of drugs depends on how well the compounds can modulate the primary target molecule, a process referred to as target engagement (TE).^3,4^ It is of great value for successful drug development if TE can be ascertained in physiologically relevant tissues.^5,6^ Analysis of purified proteins by thermal shift assays (TSAs) takes advantage of the biophysical principle that binding of ligands can stabilize or sometimes destabilize a target protein subjected to a heat challenge.^7,8^ By incubating proteins at variable temperatures, it is often possible to observe that the addition of a drug that binds a given protein causes a shift for that protein in the melting (denaturing) temperature, that is, the temperature that reduces the relative abundance by one half (*T*_m_),^7−10^ with a low rate of false positives. Affinities between drugs and target proteins ranging from pM to mM can be detected, and the extent of the shift of denaturation temperature (Δ*T*_m_) frequently correlates with drug-target affinity.^7−10^ Also, other factors such as the overall protein size and the binding to high- or low-temperature melting domains within the protein may influence protein unfolding thermodynamics.^7−10^ TSAs have been proven to be instrumental in drug discovery, and they have been used extensively in both academia and industry for characterizing drug interactions with purified proteins. The cellular thermal shift assay (CETSA) is the first broadly applicable technique for TE studies directly in cells and tissues.^11,12^ The CETSA technology builds on the observation that proteins in cells precipitate after heat-induced unfolding, and melting curves can be generated by quantifying the remaining soluble proteins after treatment at increasing temperatures or drug concentrations. As for TSA using pure proteins, ligand-stabilized proteins in complex biological samples typically yield shifts in CETSA melting curves. The assay therefore enables TE studies in cellular contexts, providing relevant information about target potency and phenotypic effects during discovery of drugs and chemical probes.^11−13^ Also, the CETSA has recently been demonstrated as a powerful method for monitoring changes in interactions of proteins with other physiological ligands such as other proteins, nucleic acids, and metabolites, and the approach provides novel biomarkers for cellular processes and downstream effects of drug action.^12^ The CETSA is highly suitable for analysis of soluble protein targets, including large protein complexes, but less efficient for membrane proteins.^14,15^

At present, the primary readout methods for the CETSA are techniques that evaluate individual target proteins such as Western blotting^16^ and antibody-based AlphaScreens and related methods^17^ or alternatively mass spectrometry (MS) for analysis on the proteome level.^18^ Western blot analyses are limited with respect to throughput and multiplexing. AlphaScreens enable high-throughput screens of the effects of large sets of compounds but only on single proteins. Imaging implementations of the CETSA can in special cases be used with a smaller number of cells but are limited in practice by the requirement that the proteins give very high-contrast melting curves.^19,20^ MS on the other hand enables studies of intracellular drug binding and downstream effects in the context of proteome-wide measurement for assessing drug safety and efficacy.^18,21−23^ However, typical MS data sets often miss ensembles of proteins of specific interest, and none of the current approaches allows high-throughput, multiplex analysis of a specific targeted set of proteins in samples with limited numbers of cells such as clinical sample materials.^13,24−26^

We reasoned that affinity-based proximity extension assays (PEAs), which allow parallel quantification of large sets of specific proteins in small sample aliquots, could prove a valuable middle ground in CETSA applications. PEAs offer both high sensitivity and specificity in a multiplex format, suitable to analyze sets of 96 proteins and controls at high sensitivity, using as little as 1 μL of plasma or lysates of tissues^27−29^ or even of single cells.^30,31^ A homogeneous PEA depends on target protein binding by oligonucleotide-conjugated antibodies. When a pair of PEA probes binds their target protein, the attached oligonucleotides are brought in proximity and their free 3′ ends can hybridize with each other, initiating a polymerization reaction upon addition of a DNA polymerase.^28^ Thereby, DNA reporter molecules are formed for each detected protein with no need for washes or separations followed by detection via quantitative real-time PCR or by DNA sequencing. The reactions are designed so that only cognate pairs of antibodies can give rise to detectable reaction products, preserving detection specificity in multiplex assays. The ability of the PEA to use very small amounts of samples and the suitability for most of the sample types are particularly valuable for CETSA applications where limited sample volumes and a requirement for rapid assay turn-around render CETSA-MS analysis unsuitable. Accordingly, the CETSA-PEA has the potential to allow testing sets of drugs in numerous biomedically relevant samples for their effects on targeted sets of proteins during drug development and in clinical care.^11−26^

**Figure 1:**
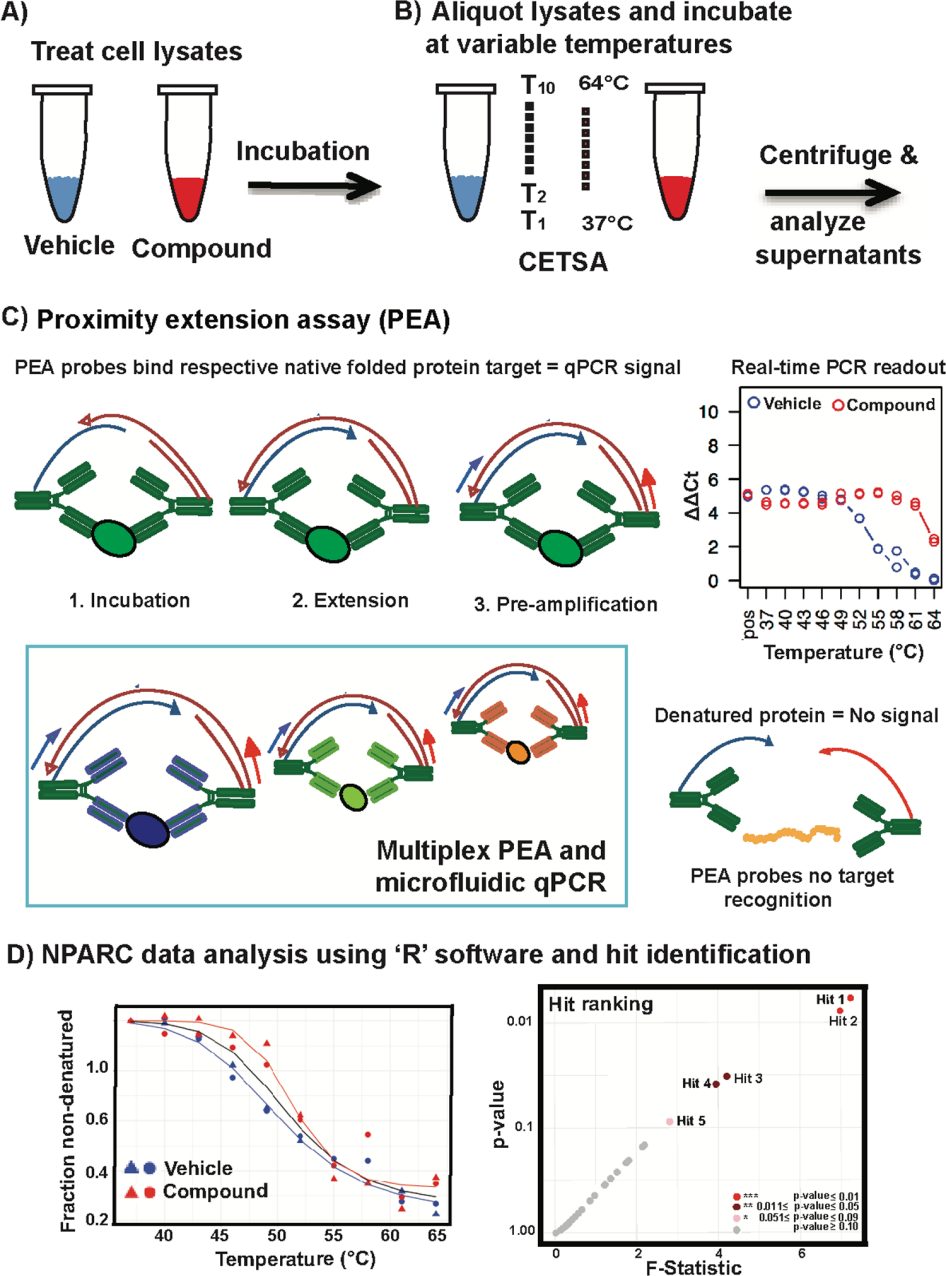
Schematic illustration of the cellular thermal shift assay (CETSA) with readout via the proximity extension assay (PEA). Representation of workflows; lysate incubation with the drug, heat treatment, centrifugation, and protein analysis of the supernatant via the PEA. (A) Cell lysates are incubated either with or without drugs and aliquoted into PCR tubes. (B) The treated aliquots are incubated at one of 10 different temperatures in a gradient PCR machine followed by removal of the precipitated protein fraction by centrifugation. (C,D) The supernatants are analyzed by the multiplex PEA. The raw data from the real-time PCR are log 2 Ct values and the CETSA-PEA data analyzed using “R” software with an open-source software implementation of the nonparametric analysis of response curves (NPARC) as developed by Childs et al.^38^ Protein stability profiles for compound-treated samples (red) and control (blue) conditions are compared and scored for hit identification based on the *p*-value and the *F*-statistic obtained from NPARC’s criterion metrics for the total area of the melt curve changes.

In this study, we evaluated CETSA analyses by multiplex PEA reactions for 67 proteins; 29 of these overlapped with a set of 6479 proteins identified by MS in the same K-562 cells. We demonstrated the CETSA-PEA approach in lysates from a human cancer cell line treated with three ATP-competitive kinase inhibitors (Figure 1). Treatment of cells with the kinase inhibitor staurosporine resulted in melting curves with concordant thermal shifts when read out by MS and the PEA. Of the 29 proteins analyzed by both MS and the PEA, good correlations of CETSA results were observed for 23 proteins, and four exhibited moderate correlation, while the correlation for two of the proteins was poor.

## Experimental Section

### CETSA in Cell Lysates

The human myeloid leukemia cell line K-562 (ATCC no. CCL-243) was used for all experiments. Approximately 40 million cells/mL were lysed by three cycles of freeze-thawing using liquid nitrogen and a heat block thermostated at 20 °C. The lysates were then clarified by centrifugation at 20,000*g* for 20 min at 4 °C followed by collection of the soluble fraction. The cell lysates were divided into aliquots, treated with 20 μM dasatinib (Sprycel, Cell Signaling), gefitinib (Iressa, Cell Signaling), or staurosporine (Cell Signaling & Sigma Aldrich) in 1% DMSO, or with DMSO alone, and incubated at room temperature for 10 min. After incubation, lysates were aliquoted into PCR tubes, 100 μL per tube. The lysates were heated for 3 min in a gradient PCR machine to one of 10 different temperatures differing by 3 °C intervals from 37 to 64 °C. The fraction of proteins precipitated by this heat treatment was removed by centrifugation at 20,000*g* for 20 min at 4 °C, and 80 μL of the supernatants was transferred to 96-well plates.

### PEA Analysis

CETSA cell lysates at 40 million cells/mL were diluted in the lysis buffer (HBSS buffer + HALT) to a concentration of 5000 cells/μL for PEA analysis. Multiple pairs of PEA probes were added to the cell lysates in microtiter wells at a final probe concentration of 100 pM each. The probe mixture (3 μL) was combined with 2.1 μL of incubation solution and 0.3 μL of stabilizer solution (Olink Proteomics) and with 1 μL of the cell lysate or 1 μL of lysis buffer alone, serving as a background control. The PCR plates were agitated gently, briefly centrifuged, sealed, and incubated at +4 to +8 °C overnight (approximately 20 h). After incubation, the reactions were spun down for a minute at room temperature, and 96 μL of a probe extension mixture was added to each well. The PEA extension mixture contained 0.5 μL of a PEA enzyme, 0.2 μL of a PCR polymerase, 10 μL of PEA solution (Olink Proteomics), and 85.3 μL of purified water. The plates were gently mixed, briefly centrifuged for 1 min at room temperature, and then placed in a preheated PCR machine. The following PEA program was run: oligonucleotide extension at 50 °C for 20 min followed by pre-amplification of the extension products via a universal primer pair at 95 °C for 5 min and then 17 cycles of 95 °C for 30 s and 60 °C for 1 min. The pre-amplified DNA reporter molecules from multiplex detection reactions were then individually decoded and quantified by real-time PCR with PCR primers specific for the DNA reporters for each of the investigated proteins. The individual amplification reactions were performed using a 96.96 Dynamic Array integrated fluidic circuit (IFC) on a Biomark HD system (Fluidigm) according to the manufacturer’s instructions. Each pre-amplified sample (2.8 μL) was mixed with 7.2 μL of the detection mixture in a new 96-well plate. The detection mixture contained 5.0 μL of detection solution, 0.071 μL of the detection enzyme, 0.0028 μL of a PCR polymerase (Olink Proteomics), and 2.1 μL of purified water. A mixture (5.0 μL) from each sample with the detection mixture was transferred into primed 96.96 Dynamic Array IFC right inlets and 5.0 μL of the primer plate in left inlets. The 96 primer pairs were designed for amplification of the target-specific DNA reporter molecules formed in the PEA reactions. The chips were run using the Olink Protein Expression 96×96 program (50 °C, 120 s; 70 °C, 1800 s; 25 °C, 600 s; 95 °C, 300 s) and (95 °C, 15 s; 60 °C, 60 s) × 40 cycles. The PEA data extracted from the Fluidigm Biomark instrument are Ct values proportional to the log 2 of the target protein concentrations. See the Supporting Information for the LC–MS/MS analysis and NPARC workflow.

## Results and Discussion

To evaluate multiplex PEA readout for CETSA analysis, we applied two exploratory, noncommercial PEA panels developed in collaboration with Olink Proteomics and targeting a total of 67 distinct proteins (Table S6 and Supporting File 1). These panels were established for analyzing protein expression in cell signaling pathways in lysates from single cells,^30^ and they were therefore useful to investigate target engagement by low-molecular-weight kinase inhibitors in lysates from small numbers of cells. Concentrations of up to 96 proteins and controls can be read out for 96 1 μL aliquots of cell lysates along with controls, using a microfluidic real-time instrument as applied here, and an even higher throughput is possible via next-generation sequencing (www.olink.com). The technique is applicable for a broad range of proteins, and the company is rapidly expanding their repertoire from the present 1500 proteins. MS provides a powerful means to investigate target engagement by drugs using the CETSA and for off-target profiling, both in the context of repurposing established drugs and to avoid adverse events by new entities.^14,16−22^ We reasoned that this ability of MS to investigate whole proteomes might for some applications be balanced against PEA’s superior sensitivity and convenience by targeting specific sets of proteins of interest in small sample aliquots.^29−33^ We selected as a cell model system the human K-562 lymphoblast cell line treated with three ATP-competitive kinase inhibitors: the clinical cancer drugs dasatinib and gefitinib, both having narrow target specificity, and the preclinical pan-inhibitor staurosporine. The responses of a K-562 cell lysate to treatment with staurosporine and dasatinib were previously investigated through the CETSA with MS detection by Savitski et al.^18^ To allow a direct comparison of MS-CETSA with PEA data, using the same sample preparation and conditions, new MS data sets were collected.

**Figure 2:**
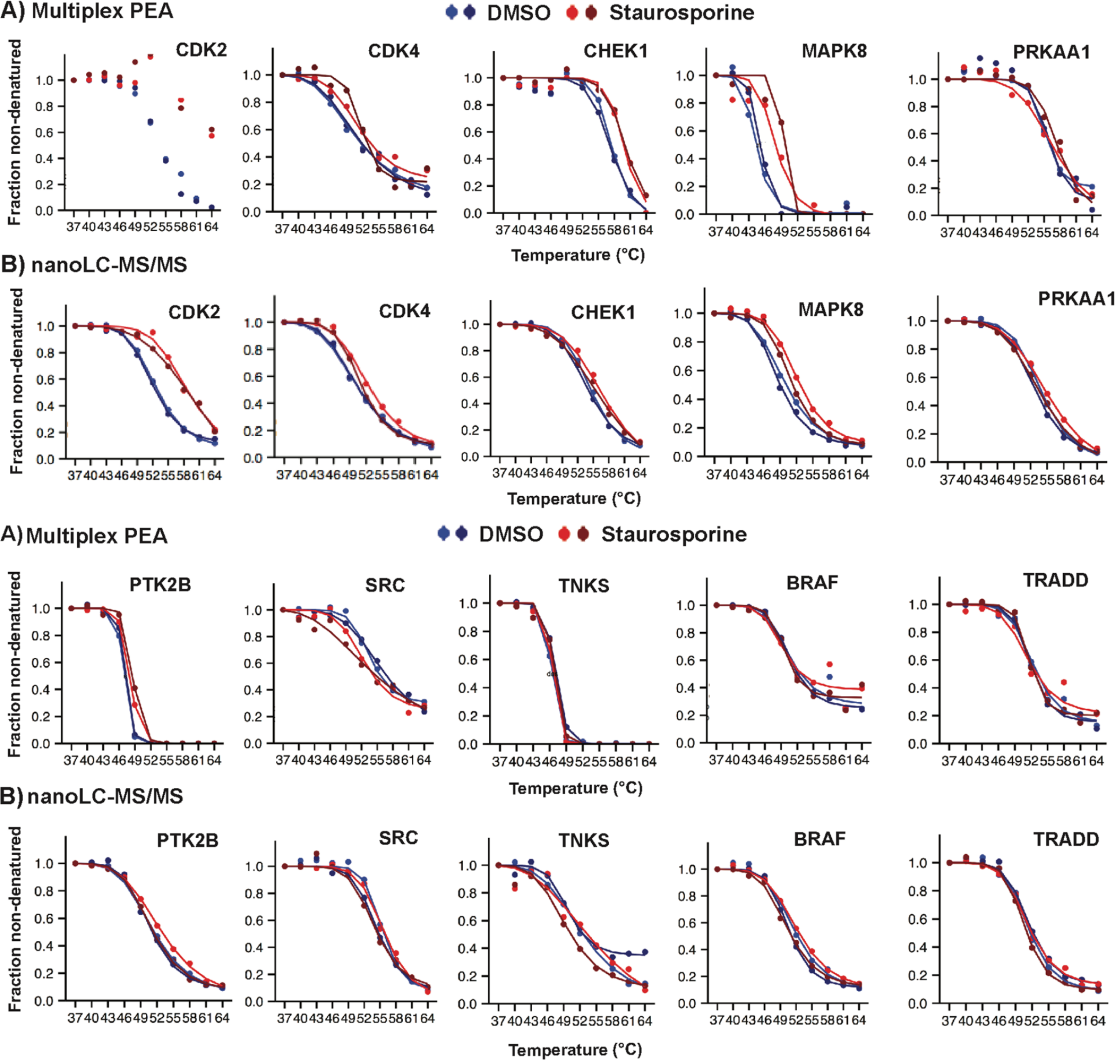
Comparison of the CETSA with PEA or MS readout. K-562 cell lysates were treated with the vehicle or 20 μM staurosporine followed by incubation at different temperatures and detection of the remaining soluble proteins. Supernatants obtained after centrifugation were analyzed in duplicate either by the multiplex PEA or nanoLC-MS/MS. (A,B) The four melting curves for each protein represent two replicates for the staurosporine-treated samples (red and dark red) and for the DMSO (vehicle)-treated controls (blue and dark blue). (A) PEA results and (B) MS results for eight known target kinases exhibited concordant melting curves for CDK2, CDK4, CHEK1, MAPK8, PRKAA1, PTK2B, SRC, and TNKS and for the kinases BRAF and TRADD that are known to be nontarget proteins for staurosporine and that were unaffected by the drug as seen using either readout. The plots for the PEA and MS were generated using an in-house script developed in “R” and normalized using the mineCETSA package. The software failed to fit the PEA data for CDK2 to the model (see Supporting Information Method S6).

A panel of 67 PEA assays had been previously evaluated by Darmanis et al. by screening against cell lysates from 1, 10, 100, and 1000 cell equivalents of several cell lines, including K-562, and by initial screening for cross-reactivity among a pool of recombinant proteins (Supporting File 1).^30^ Among the proteins targeted here, 18 were detectable by the PEA even at the level of single K-562 cells (Supporting File 1),^30^ and a majority of PEA assays used in the CETSA screen were sensitive to low numbers of K-562 cells. For our experiments here, we used 5000 cell equivalents of K-562 cell lysates for each multiplex PEA analysis. As an initial proof-of-concept CETSA-PEA experiment, we screened K-562 cell lysates treated with the kinase inhibitor staurosporine at 20 μM or with the vehicle (DMSO) serving as a control (Figures 1 and 2). Staurosporine is a broad-spectrum ATP-competitive kinase inhibitor that interacts at medium to high affinity with many kinase proteins.^34,35^ Sixty-seven proteins were targeted by the PEA, including 16 known target proteins for staurosporine (Tables S1 and S4). We validated the CETSA-PEA method by comparing the results for a selected set of proteins with those obtained by quantitative mass spectrometry (LC–MS/MS) for the same treated samples (Figure 2).

We compared results for PEA and MS readout of the CETSA using the mineCETSA package, written in R software.^36,37^ Staurosporine treatment of cell lysates yielded reproducible thermal shifts with good correlation between PEA and MS readout of CETSA experiments for known target proteins such as CDK2, CDK4, CHEK1, MAPK8, PRKAA1, PTK2B, SRC, and TNKS (Figure 2). Examples of CETSA melting curves for proteins that were unaffected by staurosporine treatment are shown for the nontarget proteins BRAF, TRADD, ATR, AKT2, BCL2L1, CASP8, CASP9, FADD, IKBKB, MEN1, PLCG1, PTPN11, SIRT1, SMAD4, and XIAP (Figure 2 and Figure S4).

**Figure 3:**
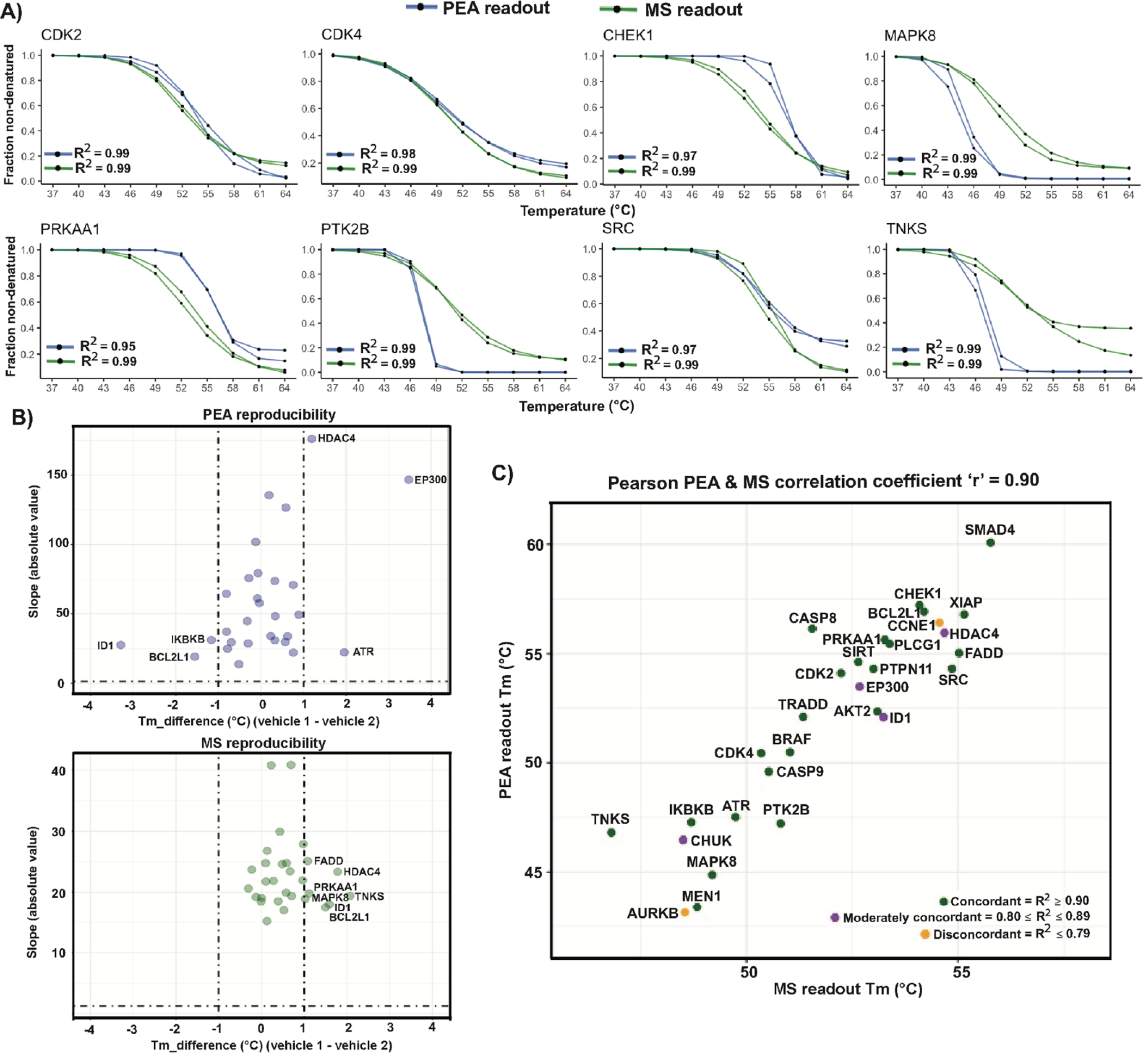
Quantitative correlation of PEA and MS readout for the CETSA. Cell lysates were treated with the vehicle, and both readouts were performed in duplicate. (A) The correlation between results from the two readout methods is illustrated by examples of melting curves for CDK2, CDK4, CHEK1, MAPK8, PRKAA1, PTK2B, SRC, and TNKS protein data for DMSO-treated control samples. The data were normalized against the median values of total soluble protein levels for each detection method. The two technical replicates for the PEA were plotted with blue lines and for MS with green lines. The *R*^2^ values (means of duplicates) indicate the goodness of fit for the plotted melting curves. (B) The volcano plots represented the distribution of the proteins’ melting point differences versus the steepest slope values from the melting curves of two control experiments as a measure of assay reproducibility. Results for the PEA are shown in blue in the top panel, and MS results are shown in green in the bottom panel. (C) Comparison of melting temperatures (*T*_m_) for the melting curves in assays recorded via MS (*X* axis) and the PEA (*Y* axis). The plots for each protein were colored according to the concordance of the two readouts as reflected in the *R*^2^ values. The Pearson correlation coefficients measured between PEA readout and MS readout of “*r*” = 0.90 for all proteins investigated by both readouts indicate the strength of their linear relationship.

The data were normalized against the median levels of the total soluble protein for each detection method. The correlation between the two readouts was estimated by *R*^2^ values indicating the goodness of fit for the two plotted melting curves (Table S2 and Figure S6). The correlation between the PEA vs MS detection was defined as concordant (*R*^2^ value ≥ 0.90), moderately concordant (0.80 ≤ *R*^2^ ≤ 0.89), or discordant (*R*^2^ ≤ 0.79) (Table S2 and Figure S6). In all, good correlation of PEA and MS readout of CETSA results was seen for 23 of the 29 proteins (*R*^2^ values ≥ 0.90; Figure 3A–C and Figures S4 and S6). Four of the proteins yielded results that were moderately correlated (0.80 ≤ *R*^2^ ≤ 0.89; CHUK, EP300, HDAC4, and ID1; Figures S5A and S6), while results for two targets were poorly correlated (*R*^2^ ≤ 0.79; AURKB and CCNE1; Figures S5B and S6). We investigated assay reproducibility by plotting melting point (*T*_m_) differences between the two replicate vehicle data sets versus the maximal slope values for PEA and MS melting curves (Figure 3B). Higher *T*_m_ differences between replicates indicate lower reproducibility. We observed absolute *T*_m_ differences of greater than 1 °C in the CETSA for 6 proteins using the PEA and for 7 with MS detection among those 29 proteins for which readout data was available both via the PEA and MS (Figure 3B). We further evaluated the correlation of the *T*_m_ values for individual proteins recorded via the PEA and MS according to their *R*^2^ values (Figure 3C). The Pearson correlation coefficient “*r*” of 0.90 indicates a strong linear relationship between results using the two readout methods (Figure 3C).

For a few proteins, CHUK, EP300, and HDAC4, measurements remained at plateau levels by the CETSA-PEA even after heating at the highest temperatures, while the same proteins were fully melted at higher temperatures according to the CETSA-MS data (Figures S5 and S6). These instances may reflect that even after treatment at the highest temperatures, the concentration of the remaining proteins in solution exceeded the dynamic range for measurement by the PEA. The melting curves by the CETSA-PEA for the known staurosporine targets AURKB and CCNE1 differ in shape from those for MS (Figure S5B). One possible explanation is that the two assays preferentially register different forms of the proteins, e.g., splice variants or post translationally modified forms.

We observed that CETSA-PEA results for CDK2, CHEK1, AKT2, CASP8, PRKAA1, and SRC shifted dramatically upward around 50 °C, indicating that more of those proteins were detected after heating the samples to higher temperatures (Figure 2A and Figures S4 and S6). The PEA assay might in these instances be influenced by whether the target proteins are in complex with other proteins, nucleic acids, or metabolites while MS detection identifies all forms of the protein. It cannot be excluded, however, that in some cases, the differences between the melting curves recorded by the PEA compared to those by MS may also be due to cross-reactivity of these PEA reactions for other, noncognate proteins^7,8^ in these only partially validated PEA reactions. For unknown reasons, analysis of the proteins BRAF, CDK2, CDK4, MAPK8, PTK2B, and TNKS by the CETSA-PEA seemed to reflect a greater susceptibility to thermal denaturation compared to results of MS analyses of the corresponding samples (Figure 3A and Figure S6). Saturated PEA detection signals were seen for some proteins, such as CHUK, IKBKB, and MEN1, consistent with the fact that lysates from 5000 cells were used throughout, although many assays could detect proteins at the levels of single cells.^30^

**Figure 4:**
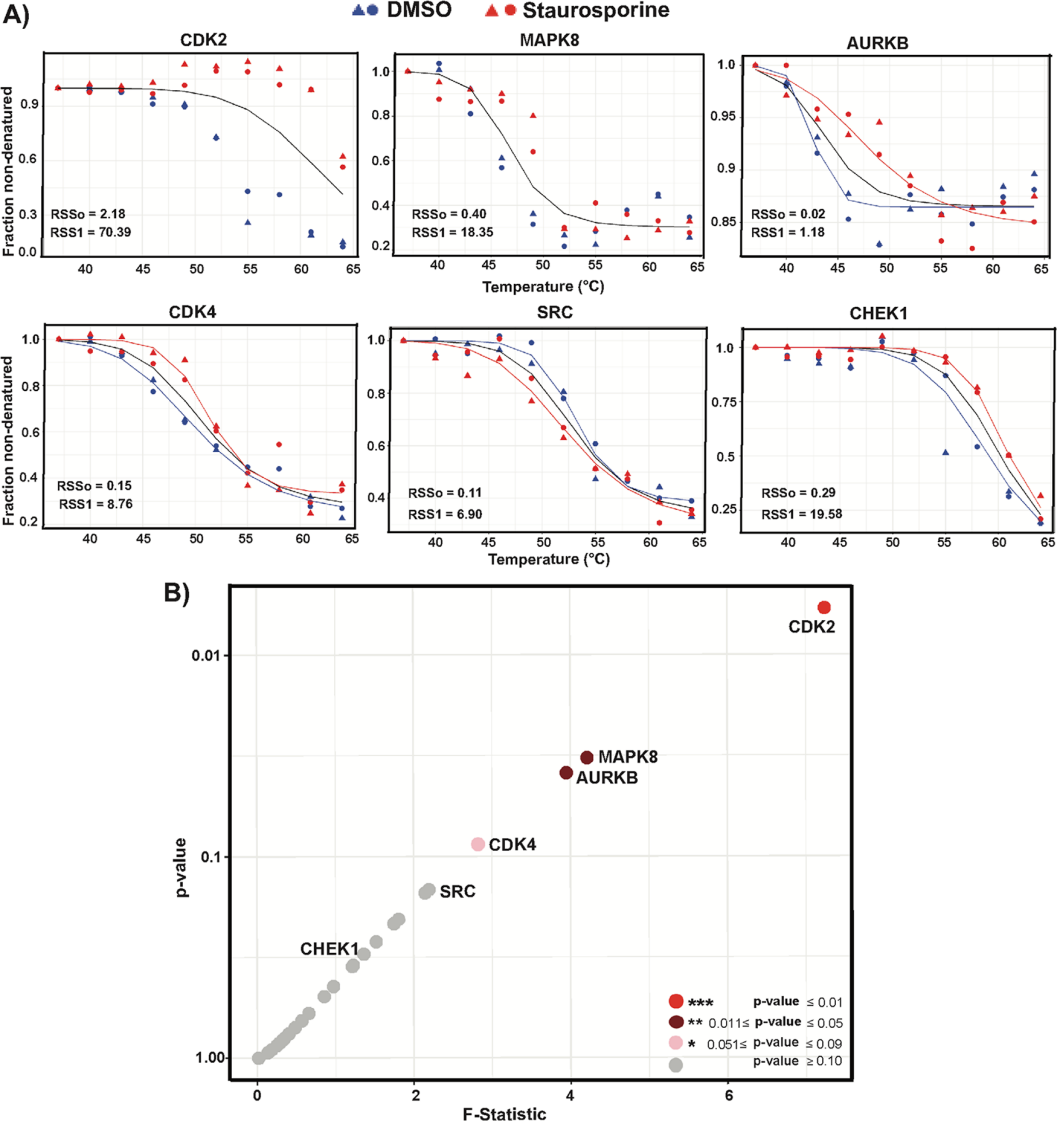
Computed test statistics for evaluation of protein stabilization by staurosporine in CETSA experiments in K-562 lysates, read out by the PEA and with ranking of hits for 29 targets. (A) Principles of NPARC analysis of the significance of protein stabilization as seen in melting curves. Significance of differences of thermal shift profiles for samples treated with staurosporine (red) or the vehicle (blue) is scored using null metrics (in black), as described by Childs et al. Fit of the null model, i.e., no treatment effect (black line). For CDK2 and MAPK8, an alternative model with separate fits for the two data sets could not be obtained using the given starting parameters. Instead, a single null fit curve is displayed, representing a combination of the results for treatment with the drug and the vehicle. Results for AURKB, CDK4, SRC, and CHEK1 have been fitted with the null and the alternative model with separate curves for the treated (red) and the vehicle condition (blue). The residual sum of square (RSS) values serve as indicators of the goodness of fit of the null and alternative models. (B) Scatter plots for the investigated proteins, representing hit identification with *F*-statistics plotted versus *p*-values for protein melting curves being significantly shifted by treatment with staurosporine compared to the vehicle. The melting curve for the protein CDK2, found to be highly significantly changed, is shown in red circles. MAPK8 and AURKB displayed moderately significant thermal shifts and are shown in dark red circles, while CDK4 had low effect size and is shown as a pink circle. Other investigated kinases and nonkinases failed to display significant shifts and are shown as gray circles.

We analyzed the significance of drug-dependent changes of the CETSA results by applying nonparametric analysis of response curves (NPARC) in R software, as developed by Childs et al.^38^ NPARC’s *F*-statistic analysis directly uses information from replicates that makes fewer assumptions on the data than the melting point (*T*_m_) estimation (the temperature of the half-maximum relative abundance).^38^ Changes of melting curves were scored using null metrics for the *p*-value and *F*-statistic criteria to determine the significance of thermal shifts (see Method S6 for the NPARC analysis workflow).^38^ PEA analysis showed that staurosporine induced significant thermal shifts for the protein kinases CDK2 and AURKB and for the moderately expressed MAPK8 (Figures 2A and 4A and Table S3). Using the CETSA-PEA, we identified CDK2 as the main hit with significantly shifted melting curves (*p*-value ≤ 0.01). Borderline significant thermal shifts were seen for MAPK8 and AURKB (*p*-value ≤ 0.05). CDK4 and CHEK1 underwent small shifts toward stabilization, while a very small shift toward destabilization was observed for SRC by PEA detection (Figures 2 and 4). NAPRC analysis revealed low effect size for CDK4 (*p*-value ≤ 0.09), and CHEK1 kinases displayed higher *p*-values (Figure 4 and Table S3).

**Figure 5:**
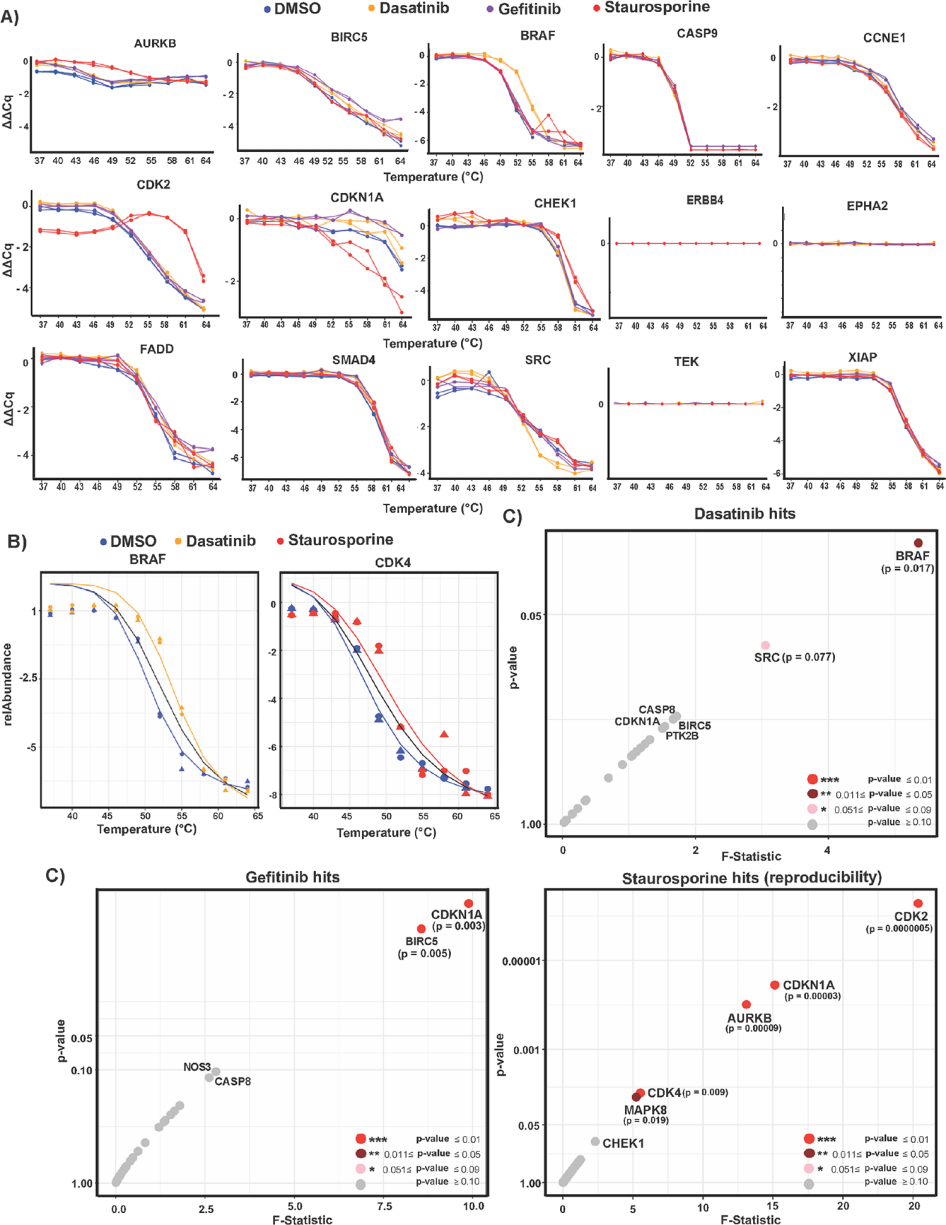
CETSA-based analysis of target engagement in K-562 cell lysates by the three kinase inhibitors dasatinib, gefitinib, and staurosporine at 20 μM compared to the vehicle DMSO and with readout via the PEA. (A) The four melting curves in each protein panel represent two independent replicates for samples treated with dasatinib (orange), gefitinib (purple), and staurosporine (red) and with DMSO serving as a control (blue). CETSA-PEA results for the proteins AURKB, BIRC5, BRAF, CDK2, CDKN1A, CHEK1, and SRC, which all exhibited thermal shifts for at least one compound each, are shown. The PEA failed to detect the ERBB4, EPHA2, and TEK proteins, consistent with the fact that K-562 cells have undetectable RNA expression of the corresponding gene (HPA; www.proteinatlas.org). These three target proteins were therefore included as a biological control. Examples of CETSA results for the proteins CASP9, CCNE1, FADD, SMAD4, and XIAP that are known to be nontarget proteins for dasatinib, gefitinib, and staurosporine are shown. (B) Applying NPARC analysis of thermal shifts of BRAF and CDK4 to fit with the null and the alternative models with and without dasatinib and staurosporine treatment. (C) Scatter plots for proteins representing the three kinase inhibitors with hits identified and ranked based on the *p*-value and the *F*-statistic number obtained from NPARC analysis. Proteins for which the kinase inhibitors staurosporine, dasatinib, and gefitinib induced protein stability changes as recorded via the PEA were evaluated as potential hits according to the *p*-values for their thermal shifts.

We used the CETSA-PEA to measure the effects of the clinical kinase inhibitors, cancer drugs dasatinib and gefitinib and the preclinical compound staurosporine, all at 20 μM with DMSO as a negative control (Figure 5A and Figure S7). Comparing melting curves for known target proteins versus nontarget proteins by an added drug as analyzed by the PEA, we noted a clear trend toward thermal stability shifts for the known dasatinib target protein BRAF and staurosporine targets AURKB, CDK2, CDK4, and CDKN1A (Figure 5A and Figure S7). Since the NPARC analysis utilizes the mean and variations across all protein targets, it is essential to exclude assays with high variation that may skew the cumulative *F*-distribution in significance measurement (see Method S6). Prior to NPARC analysis of the results, we therefore removed assays that showed poor reproducibility or generated flat curves (i.e., ERBB4, EPHA2, NTRK3, PRKAA2, and TEK) to avoid getting false hits (Figure 5A and Figure S7). Conversely, as expected, nontarget proteins with well-defined melting curves such as ATR, BCL2L1, CASP8, CASP9, CCNE1, CHUK, EP300, FADD, MDM2, NOS3, PLAU, SIRT1, SMAD4, TADD, and XIAP did not exhibit any temperature shift upon treatment with the kinase inhibitors (Figure 5A and Figure S7).

Treatment of K-562 cells with dasatinib resulted in significant stabilization of the known dasatinib target protein BRAF, as reflected in higher melting temperatures by the CETSA-PEA (Figure 5A,C). A moderate leftward thermal shift of the melting curve indicating dasatinib-induced destabilization was observed for SRC at 55–58 °C (Figure 5A). This effect on SRC was detected despite a very low RNA expression for SRC in K-562 cells at 0.5 TPM (transcripts per kilobase million; Human Protein Atlas), illustrating the sensitivity of PEA detection (Table S4). A few weak dasatinib target interactions were also observed for BIRC5, CDKN1A, and CASP8 (Figure 5A and Figure S7). For the 5 known targets, the PEA does not detect EPHA1, EPHA2, ERBB4, NTRK3, and TEK consistent with the lack of expression of the corresponding mRNAs in K-562 cells (Figure 5A and Table S4).

Of the 10 previously known dasatinib targets listed in PEA analysis, increased significant thermal stability was only observed for BRAF, while no other proteins had significantly shifted melting curves (Figure 5B,C and Tables S4 and S5). Concordant CETSA results recorded via the PEA for the known targets of dasatinib AURKB, BRAF, SRC, and TNKS agreed with published data analyzed by MS (Table S4) as reported by Savitski et al.^18^ The narrow-spectrum kinase inhibitor gefitinib significantly thermally shifted for CDKN1A and BIRC5 as an unexpected off-target interaction, while no shift was recorded for the known target protein SRC (Figure 5A,C). The known gefitinib target ERBB4 could not be evaluated for lack of gene expression in K-562 cells (Figure 5A).

The results of the staurosporine analysis were highly reproducible as evidenced by the consistent shift of melting curves in replicate experiments. As expected, CDK2, CDKN1A, AURKB, CDK4, and MAPK8 all showed thermal shifts upon staurosporine treatment by the CETSA-PEA (Figure 5, Figure S7, and Table S5). In particular, the high-affinity staurosporine targets CDK2, CDKN1A, AURKB, and CDK4^18,33−35^ all underwent large, statistically highly significant thermal shifts (Figure 5, Figure S7, and Table S5). We observed good concordance between PEA and MS results for known staurosporine targets such as CDK2, CHEK1, MAPK8, PRKAA1, and SRC treated with staurosporine by comparing our PEA results with published MS results (Table S4) reported by Savitski et al.^18^ Again, discrepancy of CETSA-MS results and published results for AURKB and CDK4 could be because MS can detect all forms of the protein, while the antibodies used for the PEA might preferentially recognize specific protein isoforms or specific protein interaction states. Savitski et al. also reported a CETSA experiment where MgATP was added to a cell extract at approximately physiological ATP concentrations (2 mM), resulting in increased stability for some proteins by this endogenous ligand.^18,39^ The CETSA-PEA experiments reported here were carried out in cell extracts without physiological ATP concentrations. This may have contributed to a failure to demonstrate significant thermal shifts for some target proteins known to be expressed in K-562 cells (Table S4). In general, the CETSA with PEA detection revealed the expected protein thermal shifts, with the degree of the thermal shift influenced by drug affinity.

## Conclusions

Assays that quantitatively measure engagement by candidate drugs with their targets can help focus drug discovery programs on those compounds that reach their targets in a cellular context with minimal off-target effects. In particular, the ability to rapidly investigate drug effects on sets of proteins of interest in small sample volumes can be particularly helpful in this balancing of efficacy and toxicity. Here, we demonstrate that the combination of the CETSA with the PEA recapitulates MS results for targeted sets of proteins of interest, in a rapid and affordable procedure, suitable for application with small sample aliquots (Figure S1). The multiplex PEA reactions could also include assays for potential downstream effects on cellular processes of on- and off-targets in the form of protein interactions or modifications.^12,15^ The performance of individual PEA tests relies on the quality of the DNA-conjugated antibodies and their validation. We found similar trends for protein detection in the K-562 cells by PEA and RNA expression levels as documented in the Human Protein Atlas (Table S6). CETSA-PEA melting curves were similar to those from CETSA-MS experiments for most of the proteins. The CETSA-PEA could be applied to many more proteins by development of further assays and by optimizing antibody selection and cell numbers.

The CETSA-PEA assays demonstrated significant thermal shifts for known protein targets of the two main kinase inhibitors studied here—staurosporine and dasatinib. The approach will be particularly valuable when limited amounts of materials are available, such as in fine-needle biopsies readily available from patients with solid tumors where only a few thousand cells can be obtained.^29^ Meanwhile, the CETSA-MS provides results for a broader range of proteins where the throughput is low and typically substantial amounts of the sample material are needed. Moreover, critical proteins are sometimes missed. Accordingly, the CETSA-PEA can help in characterizing drug-TE for specific sets of proteins suspected of being involved in pharmacological or toxicological drug responses and for groups of drugs of interest. In summary, the multiplex CETSA-PEA allows convenient analyses of targeted sets of proteins in large sets of small sample aliquots, rendering the technique suitable for analyses of structure–activity relationships involving large numbers of samples, drug candidates, or both during drug development and potentially also for corresponding analyses in routine clinical care.

## Supporting Information Available

Supporting information and methods including methods, tables, and figures; experimental section and methods, brief step by step description of the workflow and analysis of the CETSA-PEA results using NPARC, kinase inhibitors, quantitative correlation of two readouts, PEA data NPARC analysis and comparison with published data, summary of results of CETSA analysis for 67 proteins, readout for CETSA and PEA reactions, total protein melt curves, examples of CETSA melting curves for proteins that were unaffected by staurosporine treatment, and PEA and MS results exhibiting some degree of discrepancy (PDF)
(Supporting File 1) PEA assay optimization and titration experiments in cell lines (XLSX)
