# Supplementary material for: Sensitive Measurement of Drug-Target Engagement by a Cellular Thermal Shift Assay with Multiplex Proximity Extension Readout
Source: Anal Chem. 2021 Jul 28;93(31):10999–1009. doi: 10.1021/acs.analchem.1c02225 (PMC8358919; doi:10.1021/acs.analchem.1c02225)
Supplement: Supplementary file 1 — ac1c02225_si_001.pdf [file ac1c02225_si_001.pdf]

Supporting Information (SI) for

Title: Sensitive Measurement of Drug-Target Engagement by CETSA with Multiplex Proximity Extension Readout

Rasel A. Al-Amin[a*], Caroline J. Gallant[a], Phathutshedzo M. Muthelo[a], Ulf Landegren[a*]

[a]Department of Immunology, Genetics and Pathology, Science for Life Laboratory, Uppsala University, SE-751 08 Uppsala, Sweden

Correspondence Authors

***Rasel A. Al-Amin -** Department of Immunology, Genetics and Pathology, Science for Life Laboratory, Uppsala University, SE-751 08 Uppsala, Sweden; orcid.org/0000-0002-0762-9034; E-mail: rasel.al-amin@igp.uu.se

***Ulf Landegren -** Department of Immunology, Genetics and Pathology, Science for Life Laboratory, Uppsala University, SE-751 08 Uppsala, Sweden; orcid.org/0000-0002-7820-1000; E-mail: ulf.landegren@igp.uu.se

Table of Contents

Methods		Page
Method S1.	Material and reagents	S3
Method S2.	Cell culture	S3
Method S3.	Design, principle and selection of PEA assay	S3
Method S4.	LC-MS/MS analysis	S4
Method S5.	Normalization of data and curve fitting	S5
Method S6.	Brief step by step description workflow of the CETSA-PEA with NPARC statistical analysis	S5-7

Tables and Figures		
Table S1.	Kinase inhibitors, structure and known kinase targets	S8
Table S2.	Quantitative correlation of mean melting temperatures (Tm) of duplicate 29 assays of untreated proteins as recorded via PEA or MS detection	S9
Table S3.	All 29 target proteins detected via multiples CETSA-PEA combined analyzed by NPARC with 20 µM of staurosporine or DMSO	S10
Table S4.	Comparison between CETSA results read out by PEA and published data analyzed via LC-MS/MS	S11
Table S5.	PEA readout and analyzed by the NPARC approach with Benjamini-Hochberg F-test p-values upon treatment with 20 µM of dasatinib, gefitinib, staurosporine or DMSO	S12-14
Table S6.	Summary of results of CETSA analysis for 67 proteins detection via multiplex PEA	S15-17
Figure S1.	Schematic description of CETSA and readout	S18
Figure S2.	PEA reactions were performed in lysates	S19
Figure S3.	Total protein melt curves obtained by CETSA as analyzed by the Bradford protein assay	S20
Figure S4.	PEA and MS results exhibited good correlation for the proteins that were unaffected by staurosporine treatment	S21-22
Figure S5.	Examples of CETSA melting curves where PEA and MS readouts results exhibited some degree of discrepancy	S23
Figure S6.	Quantitative correlation of PEA and MS readout of duplicate untreated samples for CETSA	S24-25
Figure S7.	The figure illustrates CETSA-PEA melt curves for a set of proteins by dasatinib, gefitinib, staurosporine or DMSO	S26

References S27-28

Method S1. Material and reagents

Dasatinib (Sprycel), Gefitinib (Iressa) and Staurosporine were purchased from Cell Signaling. All compounds were dissolved in DMSO (from Sigma-Aldrich) at 10 mM. Water, TBS and PBS were purified with Nalgene Rapid-Flow Filters (Thermo Scientific). DNA oligonucleotides were purchased from Integrated DNA Technologies (IDT). Oligonucleotide concentrations were measured by UV spectroscopy using a Nanodrop ND1000 spectrophotometer.

Method S2. Cell culture and drug treated supernatants

The human myeloid leukemia cell line K-562 (ATCC No. CCL-243) was grown in RPMI 1640 medium (Sigma-Aldrich) supplemented with 10% fetal bovine serum (FBS, Gibco/Life Technologies), 2 mM L-glutamine and 100 U ml^{-1} penicillin–streptomycin (Gibco/Life Technologies). Cells were grown at 37°C in a humidified air incubator with 5% CO2, and routinely sub-cultured twice a week. Short-term passaged K-562 cells were removed from the media and harvested by centrifugation, washed once in 1xPBS, and re-suspended in Hank's balanced salt solution (HBSS), including Mg^{+2}/Ca^{+2} and with the complete protease inhibitor HALT (Protease Inhibitor Cocktail, Thermo Fisher). Cells treated with the drug or with solvent control were incubated at 10 different temperatures spaced 3 degrees apart, from 37 to 64°C, followed by centrifugation to remove precipitated proteins and collection of supernatants for analysis of proteins remaining in solution by PEA or MS. The protein concentrations were determined by Bradford assay (BioRad) (see Figure S3). 7 µl per sample were transferred to each of 3 replicate plates for PEA experiments and 50 µl

Method S3. Design, principle and selection of PEA assay

PEA probes were generated by coupling the 5′ ends of one of two chemically modified oligonucleotides to each of two aliquots of a polyclonal antibody to generate pairs of PEA probes for each target.[1] The 3′ ends of the oligonucleotides on each pair of PEA probes specific for a given protein are complementary, and both oligonucleotides include protein-specific tag sequences, as well as sequences for amplification of the reporter DNA products that form in detection reactions. Oligonucleotides on cognate pairs of PEA probes, having been brought in proximity by binding the same target protein, can extend off each other to form protein-specific DNA reporters that are quantified via qPCR.[1] We selected two custom PEA panels, each including 92 proteins and 4 controls. The two different PEA panels were run in parallel. Each 96-plex panel of assays targeted 92 different proteins and four spike-in controls, serving as incubation controls (the two non-human recombinant proteins green fluorescent protein and phycoerythrin), extension control and negative control or detection control to reveal PCR background.[1-4] The extension control was used to minimize intra-plate assay variation by allowing a pair of reporter DNA sequences to produce an extension product independently of protein recognition. Nine proteins overlapped among the two panels.

Each PEA assay was initially validated with recombinant antigens for specificity, sensitivity and for cross-reactivity among a limited set of proteins. 74 protein assays were excluded due to results of assays in K-562 cells were below the limit of detection (LOD). Another 34 of the assays proved unsatisfactory by demonstrating cross-reactivity for other proteins and were therefore excluded from the analysis.

Method S4. LC-MS/MS analysis

For CETSA-MS analysis, 20 µL of the soluble protein supernatants were transferred to a PCR-plate (equal volumes for all samples). Disulfides were reduced by adding 5 mM tris(2-carboxyethyl)phosphine (TCEP), 0.3% sodium dodecyl sulphate (SDS) and triethyl ammonium bicarbonate (TEAB), followed by incubation at 65°C for 30 min. Proteins were alkylated by adding freshly prepared chloroacetamide and incubating at room temp for 30 min in the dark. Samples were then diluted using LC-MS grade water to achieve final concentrations for trypsin digestion of <0.1% SDS and 50 mM TEAB. LysC enzyme was added at a mass ratio (protease:protein) of 1:50 followed by incubation over-night at 37°C. To complete protease digestion, the incubation was continued after the addition of trypsin (1:50 final ratio). The digestion efficiency was verified to be >90% by nanoLC-MS/MS analysis. For relative quantification, samples were labeled with Tandem Mass Tag (TMT)-reagent (10-plex versions, Thermo Fisher Scientific) by incubation at room temperature for 3 hrs. Complete labeling was verified by nanoLC-MS/MS analysis before samples were pooled to generate the final TMT-labeled samples. After strong cation-exchange solid-phase extraction (Strata-XC 30 mg cartridge, Phenomenex, Torrance, CA, USA), TMT-labeled samples were fractionated for increased depth of proteome coverage using a high pH reversed phase methodology. LC-MS/MS data for protein identification and relative quantification was acquired using an OrbiTrap-based instrument (Q Exactive, Thermo Scientific). NanoLC separation was achieved using an Easyspray format 50 cm x 75 µm ID analytical column (ES803, Thermo Scientific) with sample pre-concentration on a trap column. Precursor ion MS spectra were acquired using the following parameter settings: Resolution 70 000, maximal AGC (Automated-Gain Control) 1e6, maximum injection time 100 ms over the m/z range 300-1600. Fragment MS/MS spectra were acquired for the 5 most abundant precursors in each MS scan (of charge 2+ to 6+) using the following parameter settings; Resolution 35000, maximal AGC 2e5 ions, maximum injection time 150 ms, isolation window 2.0 and a normalized collision energy (NCE) of 30. The Sequest HT algorithm as implemented in the Proteome Discoverer 2.0 software package (Thermo Scientific) was used for peptide-spectrum-matching (protein identification). Raw data spectra were matched against a total of 42076 reviewed human protein sequences of the Uniprot database (i.e. the Swissprot database including canonical and isoform protein sequences). Search parameters were set to include; tryptic enzyme specificity with ≤2 missed cleavage sites, 10 ppm and 20 mDa MS/MS precursor and fragment ion mass accuracy respectively, oxidation of methionine as variable modification and TMT-labeling (peptide N-termini and lysine residues as fixed modifications). The following criteria were set for inclusion of individual peptide-spectrum matches; High confidence as assigned by the Percolator algorithm (using q-value), XCorr-value ≥2.0 and highest-ranking proposed sequence (rank = 1). Reporter ion intensities were extracted using a mass window of 10 ppm. To export the protein quantification result data, identified proteins were filtered for so called “Master proteins” and the data exported in .txt-format for further processing using in-house scripts developed in R.

Raw MS data were matched against a total of 42,076 human protein sequences of the Uniprot database (i.e. the Swissprot database including canonical and isoform protein sequences). 6,479 proteins were identified by MS by quantifying more than one unique peptide in each of two replicates of staurosporine- or vehicle-treated samples and were included in our analysis. Of these proteins, 29 proteins were also present among the 67 proteins investigated by PEA.

Method S5. Normalization of data and curve fitting

The PEA analysis data were normalized as signal over background as follows: (A) Data were normalized with the internal spike-in extension control to control for intraplate technical variation (Ct protein – Ct extctl = Ct protein_norm). (B) The mean and standard deviations of the background values were calculated for a given assay (after extension control normalization). (C) The signals over background were calculated as (Mean_background_protein_norm - 3*stdev_background_protein) – Ct protein_norm. Negative values are reported as zero. The normalized PEA data were plotted using a script developed in the statistical program 'R'. To compare CETSA-PEA data between dasatinib, gefitinib and staurosporine vs. DMSO, the data was further normalized to control for inter-plate differences using a duplicate positive control included on each plate. The normalized PEA data were plotted together in the same graph using ggplots as applied in 'R'. The MS data were imported into the R environment (http://www.R-project.org/) and normalized using the mineCETSA package (https://github.com/nkdailingyun/mineCETSA). After normalization, the MS data were exported in order to plot PEA and MS results together using Microsoft Excel.[5-6] In order to compensate for variation (e.g. unequal sample loading) CETSA-MS data was normalized using the median ratios of the entire dataset for each duplicate observation of drug treated and control samples. In the MS data each point of the melt curves is expressed as a ratio of the protein abundance after treatment at a particular temperature, relative to the abundance after pretreatment at 37°C (the lowest of the ten temperatures assayed) and subjected to a global normalization procedure described by Savitski et al.[7] The PEA data were not normalized in the same fashion, but using results from the internal controls commonly used for PEA.[4] To generate the corresponding ratios for the PEA-data, all abundance values for a given protein were divided by the abundance value at 37°C for each protein.

Method S6. Brief step by step description workflow of the CETSA-PEA with NPARC (nonparametric analysis of response curves) statistical analysis[8]

Step 1: We normalized and transformed the PEA data to a 0 to 1 scale to represent the ratio of the protein abundance at a particular temperature relative to the detected abundance at 37°C. The transformation creates a uniform starting point to give a more accurate comparison of the observed shifts between the experiments. The normalized Ct values obtained from CETSA-PEA measurement of CDK2 in K-562 cell lysates treated with staurosporine or vehicle (Method S6 Figure A) were transformed from normalized Ct values to relative protein abundance (Method S6 Figure B).

Step 2: The transformed data were then imported into 'R' for analysis (Method S6 Figure C) using NPARC method developed for MS-CETSA analysis by Childs et al. NPARC analysis offers a statistical method for melt curve analysis based on functional data analysis and nonlinear regression. By comparing the entire curve derived from the melt curve data and accounting for the goodness of fit for the curve in an analysis method that has high sensitivity for small but reproducible effects.[8]

Step 3: From this plot (Method S6 Figure C), we fit models to determine whether there is a significant difference between the treatment groups. First, the null model fits for data from both treatment groups to a single sigmoidal curve (an average curve for all four data replicates at each data point) (Method S6 Figure D). When fitting this curve, the difference between the fitted curve and the observed data is collected as residuals (RSS_0) to be used later in determining the F-statistic. The null model assumes no difference between the two treatment groups and if

true the fitted curve accurately represents both treatment groups while having low residual values.

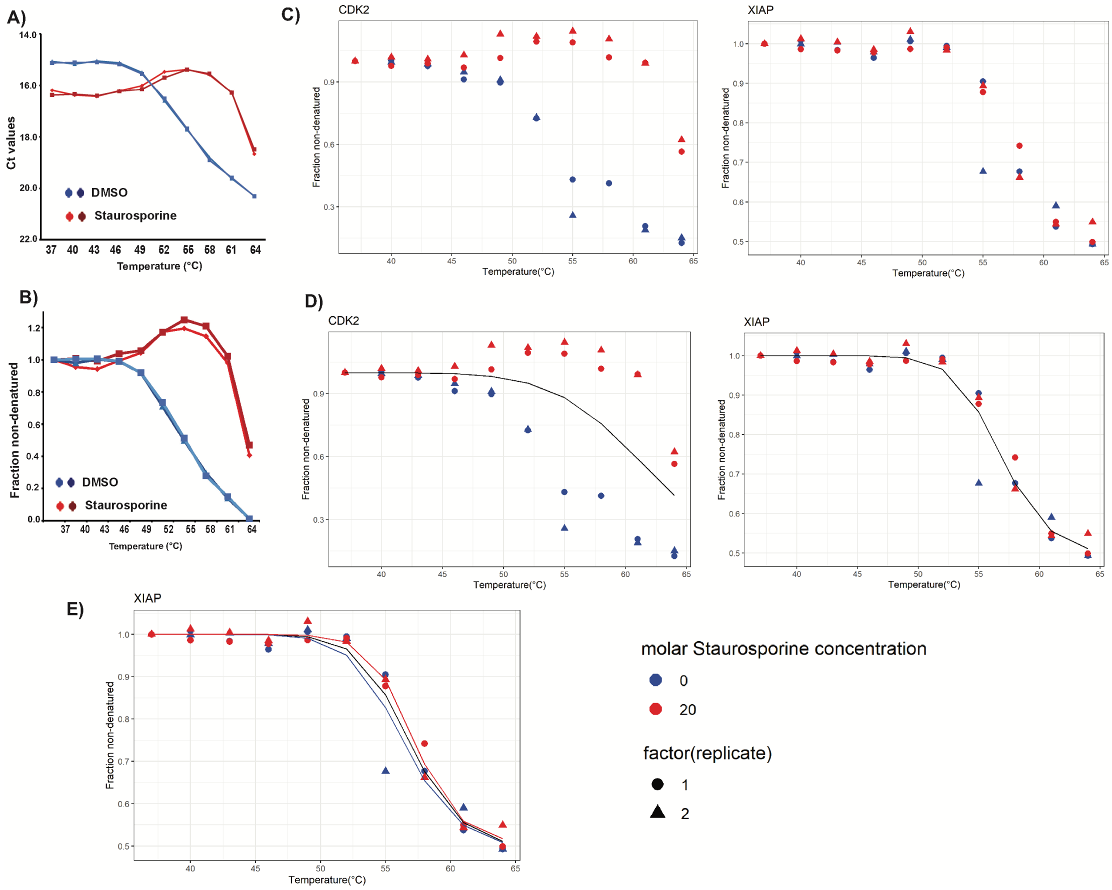

Method S6 Figure. Work-flow of NPARC analysis; the significance of drug-dependent melting curves changes of CETSA results are investigated by applying NPARC using R software. (A) CDK2 CETSA melting curve from normalized PEA data. (B) Transformed PEA melting curve data for CDK2 in samples treated with staurosporine or DMSO. (C) Plotted transformed data for CDK2 and XIAP. All replicates were individually treated with either 20 µM staurosporine or DMSO. (D) Null model fitting a sigmoid melting curve to all the data points for CDK2 and XIAP. (E). Fit of the alternative model.

Step 4: Next, we fit the alternative model, where a different model is applied to the observed data for each treatment group (Method S6 Figure E). T_m difference are either positive or negative in the two biological replicates. The smallest absolute difference between treatment and vehicle T_m is recorded. The alternative model assumes a difference between the treated and vehicle samples, motivating using separate models for the two conditions. Again, the residuals (RSS_1) are collected to be used in the *F*-statistic determination.

Step 5: To compare between the null and alternative models, the *F*-statistic is determined by measuring the difference in residual values between the null and alternative models. As the alternative models always have an equal or better fit than the null model the *F*-statistic is zero or a positive number. The resultant *F* is smaller for proteins that are not subject to any treatment effect, whereas a high value of *F* indicates a reproducible effect. The steps above are applied to all proteins, obtaining the mean and variance for each protein and treatment condition based on the observed values of RSS_0 - RSS_1.

$$F = \frac{d_2(RSS_0 - RSS_1)}{d_1 RSS_1} \quad \ldots\ldots\ldots\ldots\ldots\ldots (1)$$

For some proteins the basic model will not fit the data, as with the failed fit for the CDK2 alternative method where the treated sample does not follow a sigmoidal curve in the 1 to 0 start parameters, a function is added to the model that adjusts the start parameters slightly to obtain a fit. All proteins for which the models converged are then used in the degree of freedom estimation.

p-Values. A *p*-value for each protein is computed from its *F*-statistic and the cumulative *F*-distribution in the experiment. The cumulative F-distribution with parameters d1, d2 as described in Childs et al.[8] The multiset of p-values across all proteins was corrected for multiple testing using the method of Benjamini and Hochberg.[9] We then classify what proteins are significantly shifted by putting a threshold combined with Benjamini-Hochberg *F*-test *p*-values across replicates in an experimental design with two biological replicates.[9] We put the threshold to demonstrate different levels of significance at *p*-value ≤ 0.01 (***) for highly significant shifts, *p*-value ≤ 0.05 (**) for significant shifts and p-value ≤ 0.09 (*) as minor but not statistically significant shifts. The statistical values for all 29 protein targets that converged are placed in the Table S3 that includes the F-statistic, p-values.

Table S1. Kinase inhibitors, structure and known kinase targets[10-18]

**Compound, Other names**	**Development status**	**Structure**	**PEA assays for known targets of the compound in this study**
Gefitinib, ZD-1839; Iressa® (Astra Zeneca)	Marketed drug	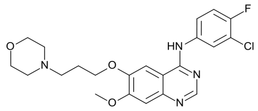	ERBB4, SRC
Dasatinib (Sprycel)	Marketed drug	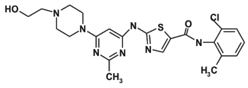	AURKB, BRAF, EPHA1, EPHA2, ERBB4, KIT, NTRK3, SRC, TEK, TNKS
Staurosporine	Research tool	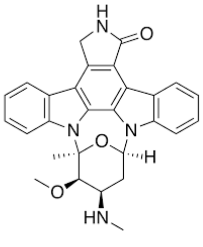	AURKB, CDK2, CDK4, CHEK1, CDKN1A, EPHA1, EPHA2, ERBB4, KIT, MAPK8, NTRK3, PRKAA1, PTK2B, SRC, TEK, TNKS

Table S2. Quantitative correlation of mean melting temperatures (Tm) of duplicate 29 assays of untreated proteins as recorded via multiplex PEA or MS detection

The melting curves were defined as concordant for R^2 values ≥ 0.90, moderately concordant when 0.80 ≤ R^2 ≤ 0.89 and discordant for R^2 ≤ 0.79

Gene name (Uniprot ID)	RNA-seq data (TPM), HPA	PEA readout Tm (°C) & R^2 value (mean of duplicates)	MS readout Tm (°C) & R^2 value (mean of duplicates)	Comments
1. **AKT2** (P31751)	51.9	52.34 & 0.96	53.09 & 0.99	Concordant
2. **ATR** (Q13535)	9.2	47.51 & 0.96	49.74 & 0.99	Concordant
*3. **AURKB** (Q96GD4)	66.5	43.13 & 0.35	48.54 & 0.99	Discordant
4. **BCL2L1** (Q07817)	161.9	56.91 & 0.92	54.19 & 0.98	Concordant
5. **BRAF** (P15056)	15.8	50.48 & 0.98	51.02 & 0.99	Concordant
6. **CASP8** (Q14790)	25.3	56.14 & 0.93	51.55 & 0.99	Concordant
7. **CASP9** (P55211)	21.1	49.60 & 0.99	50.52 & 0.99	Concordant
8. **CCNE1** (P24864)	41.1	56.41 & 0.56	54.55 & 0.99	Discordant
*9. **CDK2** (P24941)	77.7	54.09 & 0.99	52.23 & 0.99	Concordant
*10. **CDK4** (P11802)	260.8	50.44 & 0.98	50.34 & 0.99	Concordant
*11. **CHEK1** (O14757)	117.9	57.20 & 0.97	54.08 & 0.99	Correlation
12. **CHUK** (O15111)	25.2	46.47 & 0.83	48.49 &0.99	Moderately concordant
13. **EP300** (Q09472)	22.3	53.48 & 0.80	52.67 & 0.99	Moderately concordant
14. **FADD** (Q13158)	30.2	55.02 & 0.99	53.20 & 0.99	Concordant
15. **HDAC4** (P56524)	6.5	55.95 & 0.89	54.67 & 0.99	Moderately concordant
16. **ID1** (P41134)	82.7	52.08 & 0.89	53.23 & 0.99	Moderately concordant
17. **IKBKB** (O14920)	11.6	47.27 & 0.93	48.69 & 0.99	Concordant
*18. **MAPK8** (P45983)	26.5	44.87 & 0.99	49.18 & 0.99	Concordant
19. **MEN1** (O00255)	56.2	43.38 & 0.98	48.83 & 0.99	Concordant
20. **PLCG1** (P19174)	24.9	55.44 & 0.98	53.38 & 0.99	Concordant
*21. **PRKAA1** (Q13131)	14.2	55.63 & 0.95	53.26 & 0.99	Concordant
*22. **PTK2B** (Q14289)	27.8	47.21 & 0.99	50.80 & 0.99	Concordant
23. **PTPN11** (Q96EB6)	121.7	54.30 & 0.97	53.00 & 0.99	Concordant
24. **SIRT1** (Q96EB6)	19.3	54.61 & 0.97	52.64 & 0.99	Concordant
25. **SMAD4** (Q13485)	20.0	60.06 & 0.97	55.76 & 0.99	Concordant
*26. **SRC** (P12931)	0.5	54.30 & 0.97	54.85 & 0.99	Concordant
*27. **TNKS** (O95271)	13.8	46.80 & 0.99	50.89 & 0.99	Concordant
28. **TRADD** (Q15628)	6.1	52.10 & 0.99	51.33 & 0.99	Concordant
29. **XIAP** (P98170)	14.2	56.78 & 0.98	55.13 & 0.99	Concordant

Note: HPA, The Human Protein Atlas (www.proteinatlas.org). TPM, Transcripts Per Million. *known target for staurosporine.[7, 10-18]

Table S3. All 29 target proteins detected via multiples CETSA-PEA combined analyzed by NPARC approach with Benjamini-Hochberg *F*-test *p*-values upon treatment with staurosporine or DMSO

Gene name	RSS_0	RSS_1	d_1	d_2	F-Statistic	p-values
**ATK2**	0.22311098	16.4167848	3.064844	11.04063	1.25310354	0.338228439
**ATR**	0.28637629	28.2753395	3.064844	11.04063	0.01613132	0.997334497
**AURKB****	0.02549951	1.1879095	3.064844	11.04063	4.06676725	0.035292772
**BCL2L1**	0.04664676	3.0845828	3.064844	11.04063	1.80048845	0.204644177
**BRAF**	0.15963264	15.0261321	3.064844	11.04063	0.19317245	0.902074513
**CASP8**	0.17399104	16.1176629	3.064844	11.04063	0.25440348	0.860236606
**CASP9**	0.14918795	13.4651540	3.064844	11.04063	0.35604832	0.789666870
**CCNE1**	0.01283758	0.9158698	3.064844	11.04063	1.40544388	0.293241882
**CDK2*****	2.18029609	70.3996240	3.064844	11.04063	7.46240635	0.005150988
**CDK4***	0.15970303	8.7609562	3.064844	11.04063	2.91031135	0.081341588
**CHEK1**	0.29597041	19.5806709	3.064844	11.04063	1.79794707	0.205107731
**CHUK**	0.53041022	35.0900941	3.064844	11.04063	1.79802936	0.205092702
**EP300**	0.09888070	6.8390907	3.064844	11.04063	1.56312324	0.253540705
**FADD**	0.06918804	6.0268032	3.064844	11.04063	0.49913428	0.693993653
**HDAC4**	0.05972858	5.0928491	3.064844	11.04063	0.58769271	0.638815183
**ID1**	0.06285025	5.7674897	3.064844	11.04063	0.29094456	0.834845081
**IKBKB**	0.15538193	11.4028277	3.064844	11.04063	1.26603505	0.334129920
**MAPK8****	0.40794125	18.3556473	3.064844	11.04063	4.33772691	0.029488466
**MEN1**	0.21234103	19.6402172	3.064844	11.04063	0.26029425	0.856153423
**PLCG1**	0.44549279	37.1934747	3.064844	11.04063	0.67693237	0.586907415
**PRKAA1**	0.18750547	17.0159344	3.064844	11.04063	0.33455744	0.804526776
**PTK2B**	0.14135298	10.9626291	3.064844	11.04063	1.00432132	0.428602045
**PTPN11**	0.40148268	32.0028954	3.064844	11.04063	0.87967777	0.483123558
**SIRT1**	0.03383760	3.0049608	3.064844	11.04063	0.42072199	0.745586978
**SMAD4**	0.07649633	5.0030706	3.064844	11.04063	1.86026867	0.194075745
**SRC**	0.11329251	6.9032126	3.064844	11.04063	2.26101349	0.137372836
**TNKS**	0.13728969	13.0991796	3.064844	11.04063	0.14212483	0.935259531
**TRADD**	0.08850657	8.1000036	3.064844	11.04063	0.30144829	0.827533729
**XIAP**	0.05288199	4.5115784	3.064844	11.04063	0.58535732	0.640224436

Table S4. Comparison between CETSA results read out by multiplex PEA and published data analyzed via LC-MS/MS for 16 known targets for staurosporine (S) and 10 for dasatinib (D) (on- and off-target interactions)[7,10-18]

**(Drug target) Target gene name (HPA K562 RNA-seq data, TPM)**	**CETSA-PEA** (In this study)		**MS-CETSA** (Savitski et al)[7]	
	**Staurosporine** (20 µM)	**Dasatinib** (20 µM)	**Staurosporine** (20 µM)	**Dasatinib** (5 µM)
[S, D] **AURKB** (66.5)	Hit ***	MC	Does not fulfill any criteria as hit	Does not fulfill any criteria as hit
[D] **BRAF** (15.8)	MC	Hit**		Fulfills 3 criteria as hit (except no. 1)
[S, D] **CDK2** (77.7)	Hit***	MC	Hit (fulfills all 4 criteria as hit)	
[S] **CDK4** (260.8)	Hit***	Not fulfill as hit	Does not fulfill any criteria as hit	
[S] **CHEK1** (117.9)	Does not fulfill as hit	MC	Does not fulfill any criteria as hit	
[S] **CDKN1A** (4.4)	Hit***	MC		
[S, D] **EPHA1** (0.0)	ND	ND		
[S, D] **EPHA2** (0.0)	ND	ND		
[S, D] **ERBB4** (0.0)	ND	ND		
[S, D] **KIT** (3.1)	MC	MC		
[S] **MAPK8** (26.5)	Hit**	MC	Hit (fulfill all 4 criteria as hit)	
[S, D] **NTRK3** (0.0)	ND	ND		
[S] **PRKAA1** (14.2)	MC	MC	Does not fulfill any criteria as hit	
[S] **PTK2B** (27.8)	MC	MC		
[S, D] **SRC** (0.5)	Does not fulfill criteria as hit	Does not fulfill criteria as hit	Does not fulfill any criteria as hit	Does not fulfill any criteria as hit
[S, D] **TEK** (0.0)	ND	ND		
[S, D] **TNKS** (13.8)	Does not fulfill as hit	MC		Fulfills only 1 criterion as hit

Note: HPA, The Human Protein Atlas (www.proteinatlas.org). ND = No evidence of detection. TPM = Transcripts Per Million.

CETSA-PEA:

Hit*** = p-value ≤ 0.01 for highly significant shift. Hit** = p-value ≤ 0.05 for significant shift. Does not fulfill criteria as hit = Melting curve (MC) & thermal shift (TS) with p-value > 0.05. MS-CETSA hit = fulfill all 4 criteria.

MS-CETSA (Savitski et al)[7]:

Hit = fulfill all 4 criteria

1. (pVal_adj_quantiles_Expt_1<0.05 and pVal_adj_quantiles_Expt_2<0.1) or (pVal_adj_quantiles_Expt_1<0.1 and pVal_adj_quantiles_Expt_2<0.05),

2. diff_meltP_Expt_1 and diff_meltP_Expt_2 have the same sign,

3. min (diff_meltP_Expt_1, diff_meltP_Expt_2)>diff_meltP_Vehicle vs Vehicle

4. min_slope is below -0.06 in both biological replicates.

Table S5. Proteins recorded via CETSA with multiplex PEA readout and analyzed by the NPARC approach with Benjamini-Hochberg F-test p-values upon treatment with 20 µM of dasatinib, gefitinib, staurosporine or DMSO

20 top targets detected upon dasatinib treatment analyzed by NPARC approach

Gene name	RSS_0	RSS_1	d_1	d_2	F-Statistic	p-values	Adjusted p-values
**1. BRAF****	22.830100	23.568051	2.013281	14.575	5.35343631	0.01792492	0.4122731
**2. SRC***	10.851865	13.705118	2.013281	14.575	3.05404606	0.07776652	0.6707482
**3. CASP8**	13.831252	20.073490	2.013281	14.575	1.71791125	0.21378360	0.6707482
**4. BIRC5**	4.265113	6.222274	2.013281	14.575	1.67147699	0.22201520	0.6707482
**5. CDKN1A**	3.519189	5.137187	2.013281	14.575	1.66605864	0.22299898	0.6707482
**6. PTK2B**	11.256038	16.665628	2.013281	14.575	1.54076915	0.24718878	0.6707482
**7. SIRT1**	9.834302	14.619627	2.013281	14.575	1.50532718	0.25456305	0.6707482
**8. NOS3**	8.040005	12.217032	2.013281	14.575	1.31578843	0.29850296	0.6707482
**9. CAV2**	7.096501	10.875265	2.013281	14.575	1.24348080	0.31748919	0.6707482
**10. KIT**	4.124861	6.370843	2.013281	14.575	1.17748357	0.33602296	0.6707482
**11. TRADD**	11.406647	17.744714	2.013281	14.575	1.11716814	0.35404165	0.6707482
**12. CDK2**	6.570431	10.290141	2.013281	14.575	1.06123850	0.37173573	0.6707482
**13. PTPN11**	19.623386	30.816091	2.013281	14.575	1.03878911	0.37911855	0.6707482
**14. TNFRSF10B**	2.629862	4.197881	2.013281	14.575	0.90467618	0.42684931	0.7012524
**15. FADD**	10.503675	17.208879	2.013281	14.575	0.69524665	0.51567047	0.7906947
**16. SMAD4**	22.379195	38.290740	2.013281	14.575	0.35842915	0.70611206	0.9695340
**17. CHEK1**	19.090943	32.731252	2.013281	14.575	0.34294577	0.71661208	0.9695340
**18. XIAP**	18.901637	32.887512	2.013281	14.575	0.23208986	0.79712819	0.9733901
**19. ATR**	4.112308	7.228975	2.013281	14.575	0.15576128	0.85844686	0.9733901
**20. BCL2L1**	3.493049	6.145573	2.013281	14.575	0.14952190	0.86368647	0.9733901

20 top targets detected upon gefitinib treatment analyzed by NPARC approach

Gene name	RSS_0	RSS_1	d_1	d_2	F-Statistic	p-values	adjusted p-values
**1. CDKN1A****	4,152274	2,69455	1,660938	13,025	9,885802	0,003409	0,065622
**2. BIRC5***	8,798804	6,17125	1,660938	13,025	8,560333	0,005706	0,065622
**3. NOS3**	7,928366	8,565003	1,660938	13,025	2,807077	0,103583	0,673192
**4. CASP8**	16,52816	18,17755	1,660938	13,025	2,618324	0,117077	0,673192
**5. TRADD**	13,64891	16,29972	1,660938	13,025	1,791271	0,20688	0,735174
**6. ATR**	4,081434	4,948813	1,660938	13,025	1,645847	0,2301	0,735174
**7. PTK2B**	12,02804	14,75391	1,660938	13,025	1,536725	0,249572	0,735174
**8. FADD**	11,88795	14,8166	1,660938	13,025	1,388272	0,27931	0,735174
**9. KIT**	4,637292	5,803844	1,660938	13,025	1,349889	0,287677	0,735174
**10. SIRT1**	10,86742	13,814	1,660938	13,025	1,208302	0,321259	0,738895
**11. PTPN11**	21,55884	28,62145	1,660938	13,025	0,823427	0,43976	0,919499
**12. CDK2**	8,000205	10,87252	1,660938	13,025	0,623006	0,522998	0,967037
**13. CAV2**	6,967669	9,630485	1,660938	13,025	0,481302	0,594372	0,967037
**14. SMAD4**	26,07683	36,37846	1,660938	13,025	0,404447	0,638568	0,967037
**15. SRC**	8,177813	11,55944	1,660938	13,025	0,296734	0,708607	0,967037
**16. CHEK1**	24,85566	35,26747	1,660938	13,025	0,265879	0,730741	0,967037
**17. XIAP**	22,14018	31,59337	1,660938	13,025	0,219976	0,765704	0,967037
**18. BRAF**	12,97873	18,67744	1,660938	13,025	0,152132	0,82271	0,967037
**19. BCL2L1**	4,019515	5,817954	1,660938	13,025	0,106035	0,866121	0,967037
**20. TNFRSF10B**	3,660578	5,303548	1,660938	13,025	0,098348	0,873831	0,967037

Top 20 targets detected in the staurosporine treatment data analyzed by NPARC approach

Gene name	RSS_0	RSS_1	d_1	d_2	F-Statistic	p-values
**1. CDK2*** **	48.2210508	47.566714	1.907813	22.125	20.06211152	0.00001324298
**2. CDKN1A*** **	7.8999456	10.551986	1.907813	22.125	11.78355633	0.0003821854
**3. AURKB*** **	5.9316898	8.491684	1.907813	22.125	10.21771362	0.0008252086
**4. CDK4** **	27.9918013	54.154778	1.907813	22.125	4.54504641	0.02353745
**5. MAPK8** **	0.8130626	1.602978	1.907813	22.125	4.24320852	0.02905522
**6. CHEK1**	18.9309152	44.001824	1.907813	22.125	1.83885089	0.1835967
**7. CASP8**	13.0529583	32.551547	1.907813	22.125	0.92579994	0.4069102
**8. PLCG1**	22.3569842	56.129141	1.907813	22.125	0.84209933	0.4392151
**9. BRAF**	15.0479311	37.883175	1.907813	22.125	0.80793598	0.4532128
**10. NOS3**	8.8629132	22.905664	1.907813	22.125	0.48663677	0.6123887
**11. BIRC5**	5.0790716	13.15588	1.907813	22.125	0.45970124	0.6283804
**12. TRADD**	13.5443114	35.310165	1.907813	22.125	0.38201826	0.6772524
**13. FADD**	10.0746061	26.519994	1.907813	22.125	0.26665979	0.7582281
**14. TNFRSF10B**	4.0040704	10.544963	1.907813	22.125	0.26125156	0.7622976
**15. SMAD4**	26.3116141	69.796757	1.907813	22.125	0.17570478	0.8303528
**16. PLAU**	4.0459648	10.787168	1.907813	22.125	0.11628331	0.8821974
**17. ID2**	2.8078457	7.490403	1.907813	22.125	0.10963377	0.8882666
**18. XIAP**	21.3590452	57.304829	1.907813	22.125	0.04304355	0.9526598
**19. ATR**	3.4605144	9.289358	1.907813	22.125	0.03672862	0.9591796
**20. CASP9**	11.7896913	31.717105	1.907813	22.125	0.01141362	0.9864412

Table S6. Summary of results of CETSA analysis for 67 proteins in K562 lysates treated with dasatinib, gefitinib and staurosporine with detection via multiplex PEA

Note: HPA, The Human Protein Atlas (www.proteinatlas.org). TPM, Transcripts Per Million. MC, Melting curve. ND, No evidence of detection. TS, Thermal shifts.

PEA assays (Uniprot ID)	Antibody ID (R&D Systems)	HPA RNA-seq data (TPM)	Comments
**1. AKT2 (P31751)**	AF2315 (Human/Mouse/Rat Akt2 Affinity Purified Polyclonal Ab, Rabbit IgG)	**51.9**	**MC**
**2. ATR (Q13535)**	AF4717 (Human ATR Affinity Purified Polyclonal Ab, Goat IgG)	**9.2**	**MC**
**3. AURKB (Q96GD4)**	AF4006 (Human/Mouse Aurora B Affinity Purified Polyclonal Ab, Goat IgG)	**66.5**	**Hit**
**4. BCL2L1 (Q07817)**	AF800 (Human/Mouse Bcl-x Affinity Purified Polyclonal Ab, Rabbit IgG)	**161.9**	**MC**
**5. BIRC5 (O15392)**	AF6471 (Human Survivin Affinity Purified Polyclonal Ab, Goat IgG)	**103.8**	**MC**
**6. BMP7 (P18075)**	AF354 (Human/Mouse BMP-7 Affinity Purified Polyclonal Ab, Goat IgG)	**0.3**	**ND**
**7. BRAF (P15056)**	AF3424 (Human B-Raf Affinity Purified Polyclonal Ab, Goat IgG)	**15.8**	**Hit**
**8. CASP8 (Q14790)**	AF705 (Human/Mouse Caspase-8 Affinity Purified Polyclonal Ab, Goat IgG)	**25.3**	**MC**
**9. CASP9 (P55211)**	AF8301 (Human Caspase-9 Affinity Purified Polyclonal Ab, Goat IgG)	**21.1**	**MC**
**10. CAV2 (P51636)**	AF5788 (Human/Mouse/Rat Caveolin-2 Affinity Purified Polyclonal Ab, Goat IgG)	**10.2**	**MC**
**11. CCNE1 (P24864)**	AF6810 (Human Cyclin E1 Affinity Purified Polyclonal Ab, Sheep IgG)	**41.1**	**MC**
**12. CDH1 (P12830)**	AF648 (Human E-Cadherin Affinity Purified Polyclonal Ab, Goat IgG)	**0.0**	**ND**
**13. CDH3 (P22223)**	AF861 (Human P-Cadherin Affinity Purified Polyclonal Ab, Goat IgG)	**0.0**	**ND**
**14. CDK2 (P24941)**	AF4654 (Human/Mouse CDK2 Affinity Purified Polyclonal Ab, Goat IgG)	**77.7**	**Hit**
**15. CDK4 (P11802)**	AF5254 (Human/Mouse CDK4 Affinity Purified Polyclonal Ab, Goat IgG)	**260.8**	**Hit**
**16. CDKN1A (P38936)**	AF1047 (Human p21/CIP1/CDKN1A Affinity Purified Polyclonal Ab, Goat IgG)	**4.4**	**Hit**
**17. CDKN2A (P42771)**	AF5779 (Human p16INK4a/CDKN2A Affinity Purified Polyclonal Ab, Goat IgG)	**0.1**	**ND**
**18. CHEK1 (O14757)**	AF1630 (Human/Mouse/Rat Chk1 Affinity Purified Polyclonal Ab, Goat IgG)	**117.9**	**MC & TS**
**19. CHUK (O15111)**	AF3768 (Human/Mouse/Rat IKK alpha Affinity Purified Polyclonal Ab, Sheep IgG)	**25.2**	**MC**
**20. CSF3R (Q99062)**	AF-381-PB (Human G-CSF R/CD114 Affinity Purified Polyclonal Ab, Goat IgG)	**0.2**	**ND**
**21. EP300 (Q09472)**	AF3789 (Human p300 Affinity Purified Polyclonal Ab, Goat IgG)	**22.3**	**MC**
**22. EPHA1 (P21709)**	AF638 (Human EphA1 Affinity Purified Polyclonal Ab, Goat IgG)	**0.0**	**ND**
**23. EPHA2 (P29317)**	AF3035 (Human EphA2 Affinity Purified Polyclonal Ab, Goat IgG)	**0.3**	**ND**
**24. ERBB4 (Q15303)**	AF1131 (Human ErbB4 Affinity Purified Polyclonal Ab, Goat IgG)	**0.0**	**ND**
**25. FADD (Q13158)**	AF2938 (Human FADD Affinity Purified Polyclonal Ab, Goat IgG)	**30.2**	**MC**
**26. FASLG (P48023)**	AF126 (Human Fas Ligand/TNFSF6 Affinity Purified Polyclonal Ab, Goat IgG	**0.0**	**ND**
**27. FOXC2 (Q99958)**	AF5044 (Human FoxC2 Affinity Purified Polyclonal Ab, Sheep IgG)	**0.0**	**ND**
**28. GPX1 (P07203)**	AF3798 (Glutathione Peroxidase 1 Affinity Purified PAb, Goat IgG)	**0.3**	**ND**

**29. HDAC4 (P56524)**	AF6205 (Human/Mouse/Rat Histone Deacetylase 4 Affinity Purified PAb, Sheep IgG)	**6.5**	**MC**
**30. HMGA2 (P52926)**	AF3184 (Human/Mouse HMGA2 Affinity Purified Polyclonal Ab, Goat IgG)	**0.1**	**ND**
**31. HMOX1 (P09601)**	AF3776 (Human/Mouse HO-1/HMOX1 Affinity Purified Polyclonal Ab, Goat IgG)	**0.0**	**ND**
**32. HPN (P05981)**	AF4776 (Human Hepsin Affinity Purified Polyclonal Ab, Sheep IgG)	**0.0**	**ND**
**33. ID1 (P41134)**	AF4377 (Human/Mouse ID1 Affinity Purified Polyclonal Ab, Goat IgG)	**82.7**	**MC**
**34. ID2 (Q02363)**	AF4660 (Human ID2 Affinity Purified Polyclonal Ab, Goat IgG)	**5.4**	**MC**
**35. IGFBP3 (P17936)**	AF675 (Human IGFBP-3 Affinity Purified Polyclonal Ab, Goat IgG)	**0.1**	**ND**
**36. IKBKB (O14920)**	AF4535 (Human IKK beta Affinity Purified Polyclonal Ab, Sheep IgG)	**11.6**	**MC**
**37. ITGA1 (P56199)**	AF5676 (Human Integrin alpha 1/CD49a Affinity Purified Polyclonal Ab, Sheep IgG)	**0.0**	**ND**
**38. KIT (P10721)**	AF332 (Human SCF R/c-kit Affinity Purified Polyclonal Ab, Goat IgG)	**3.1**	**MC**
**39. LEP (Q6NT58)**	AF398 (Human Leptin Affinity Purified Polyclonal Ab, Goat IgG)	**0.0**	**ND**
**40. MAPK8 (P45983)**	AF1387 (Human/Mouse/Rat JNK Pan Specific Affinity Purified PAb, Rabbit IgG)	**26.5**	**Hit**
**41. MDM2 (Q00987)**	AF1244 (Human/Mouse/Rat MDM2 Affinity Purified Polyclonal Ab, Rabbit IgG)	**41.2**	**MC**
**42. MEN1 (O00255)**	AF6005 (Human Menin Affinity Purified Polyclonal Ab, Goat IgG)	**56.2**	**MC**
**43. NOS3 (P29474)**	AF950 (Human eNOS Affinity Purified Polyclonal Ab, Goat IgG)	**14.9**	**MC**
**44. NTRK2 (Q16620)**	AF397 (Human TrkB Affinity Purified Polyclonal Ab, Goat IgG)	**0.0**	**ND**
**45. NTRK3 (Q96CY4)**	AF373 (Human TrkC Affinity Purified Polyclonal Ab, Goat IgG)	**0.0**	**ND**
**46. PLAU (P00749)**	AF1310 (Human u-Plasminogen Activator/Urokinase Aff Pur Pab, Goat IgG)	**1.0**	**MC**
**47. PLCG1 (P19174)**	AF3288 (Human/Mouse/Rat PLC-gamma 1 Affinity Purified Polyclonal Ab, Goat IgG)	**24.9**	**MC**
**48. PRKAA1 (Q13131)**	AF3197 (Human/Mouse/Rat AMPK alpha 1 Affinity Purified PAb, Goat IgG)	**14.2**	**MC**
**49. PRKAA2 (P54646)**	AF2850 (Human/Mouse/Rat AMPK alpha 2 Affinity Purified Polyclonal Ab, Goat IgG)	**0.0**	**ND**
**50. PTGS2 (P35354)**	AF4198 (Human/Mouse COX-2 Affinity Purified Polyclonal Ab, Goat IgG)	**0.0**	**ND**
**51. PTK2B (Q14289)**	AF4589 (Human PYK2/FAK2 Affinity Purified Polyclonal Ab, Sheep IgG)	**27.8**	**MC**
**52. PTPN11 (Q06124)**	AF1894 (Human/Mouse/Rat SHP-2 Affinity Purified Polyclonal Ab, Goat IgG)	**121.7**	**MC**
**53. SHH (Q15465)**	AF464 (Human/Mouse Sonic Hedgehog/Shh N-Term Affinity Purified PAb, Goat IgG)	**0.0**	**ND**
**54. SIRT1 (Q96EB6)**	AF7714 (Human Sirtuin 1/SIRT1 Affinity Purified Polyclonal Ab, Sheep IgG)	**19.3**	**MC**
**55. SMAD4 (Q13485)**	AF2097 (Human Smad4 Affinity Purified Polyclonal Ab, Goat IgG)	**20.0**	**MC**
**56. SNAI1 (O95863)**	AF3639 (Human Snail Affinity Purified Polyclonal Ab, Goat IgG)	**6.3**	**MC**
**57. SOX9 (P48436)**	AF3075 (Human SOX9 Affinity Purified Polyclonal Ab, Goat IgG)	**0.0**	**ND**
**58. SRC (P12931)**	AF3389 (Human/Mouse/Rat Src Affinity Purified Polyclonal Ab, Goat IgG)	**0.5**	**MC & TS**
**59. TEK (Q02763)**	AF313 (Human Tie-2 Affinity Purified Polyclonal Ab, Goat IgG)	**0.0**	**ND**
**60. TGFB2 (P61812)**	AF-302-NA (TGF-beta 2/1.2 Affinity Purified Polyclonal Ab, Goat IgG)	**0.0**	**ND**

**61. THY1 (P04216)**	AF2067 (Human CD90/Thy1 Affinity Purified Polyclonal Ab, Sheep IgG)	**0.0**	**ND**
**62. TNFRSF10B (Q6FH58)**	AF631 (Human TRAIL R2/TNFRSF10B Affinity Purified Polyclonal Ab, Goat IgG)	**13.9**	**MC**
**63. TNKS (O95271)**	AF7116 (Human Tankyrase 1 Affinity Purified Polyclonal Ab, Sheep IgG)	**13.8**	**MC**
**64. TRADD (Q15628)**	AF2658 (Human TRADD Affinity Purified Polyclonal Ab, Goat IgG)	**6.1**	**MC**
**65. TWIST1 (Q15672)**	AF6230 (Human Twist-1 Affinity Purified Polyclonal Ab, Sheep IgG)	**0.0**	**ND**
**66. WNT2 (P09544)**	AF3464 (Human Wnt-2 Affinity Purified Polyclonal Ab, Goat IgG)	**0.0**	**ND**
**67. XIAP (P98170)**	AF8221 (Human/Mouse/Rat XIAP Affinity Purified Polyclonal Ab, Goat IgG)	**14.2**	**MC**

Figure S1.

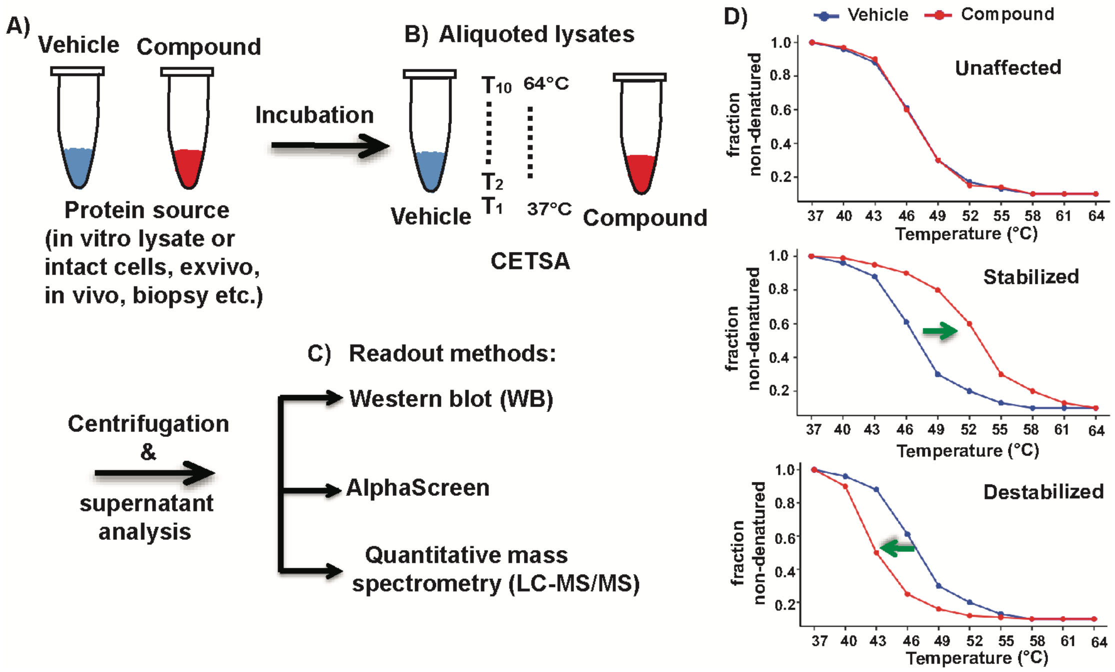

Figure S1. Schematic description of the thermal shift assay and readout for CETSA.[7,19-22] (A) Drug incubation: the cell lysates are incubated with drug or control, followed by aliquoting into PCR tubes. (B) Heat treatment: aliquoted samples are subjected to heating at either of ten different temperatures in a gradient PCR machine and the denatured and precipitated protein fraction is removed by centrifugation. (C) Protein analysis: soluble protein fractions are commonly analyzed by western blot, AlphaScreen or LC-MS/MS, and herein using PEA. (D) The CETSA assay measures drug-target binding as reflected in drug-induced stabilization or sometimes destabilization of the molecular target. Rightward shifts of a melting curve indicate compound-induced stabilization of the proteins while leftward shift represents destabilization.

Figure S2.

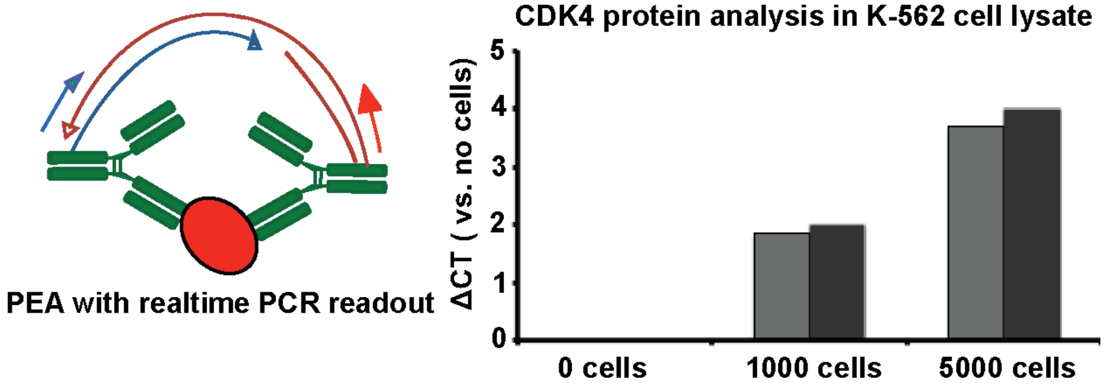

Figure S2. PEA reactions were performed in lysates from 1000 or 5000 K-562 cells. The grey and black bars represent duplicate observations. The data were normalized for delta cycle threshold (Ct) by subtracting sample Ct from Ct of negative controls with no lysate added.

Figure S3.

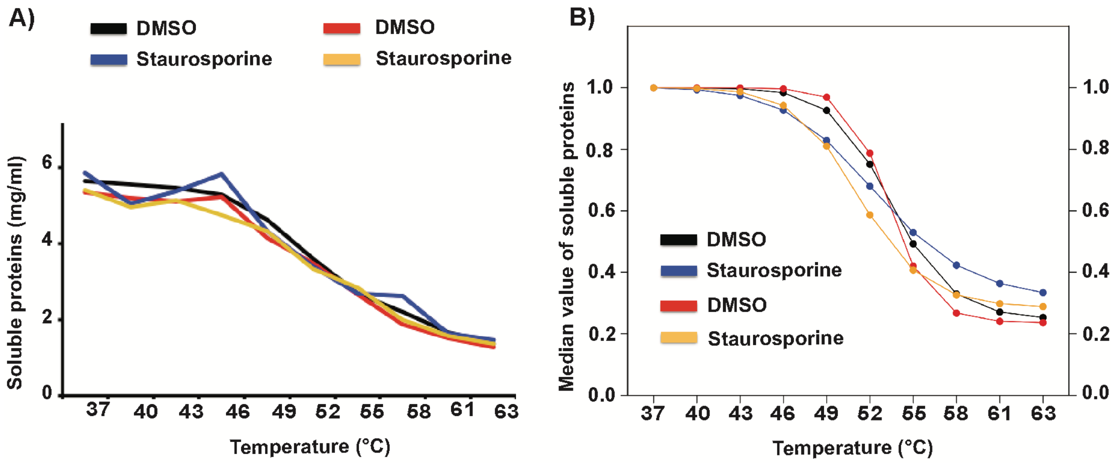

Figure S3. Total protein melt curves obtained by CETSA as analyzed by the Bradford protein assay. MS. K-562 cell lysate was treated with staurosporine or with DMSO as a control before heating aliquots at ten different temperatures, followed by removal of precipitated proteins by centrifugation. (A) The protein concentrations after heat treatment were determined in two biological replicates for both experimental groups (control and drug treated) using the Bradford protein assay. (B) The same analysis but evaluating effects on all 6,479 proteins detected by MS. MS successfully analyzed 6,479 proteins and the data were normalized against the median levels of soluble protein. The total protein abundances for each point of the melt curve are expressed as a ratio of the abundance at the lowest temperature (which is set to 1). The results in (A) and (B) demonstrate no overall effects on protein sensitivity to thermal denaturation by staurosporine.

Figure S4.

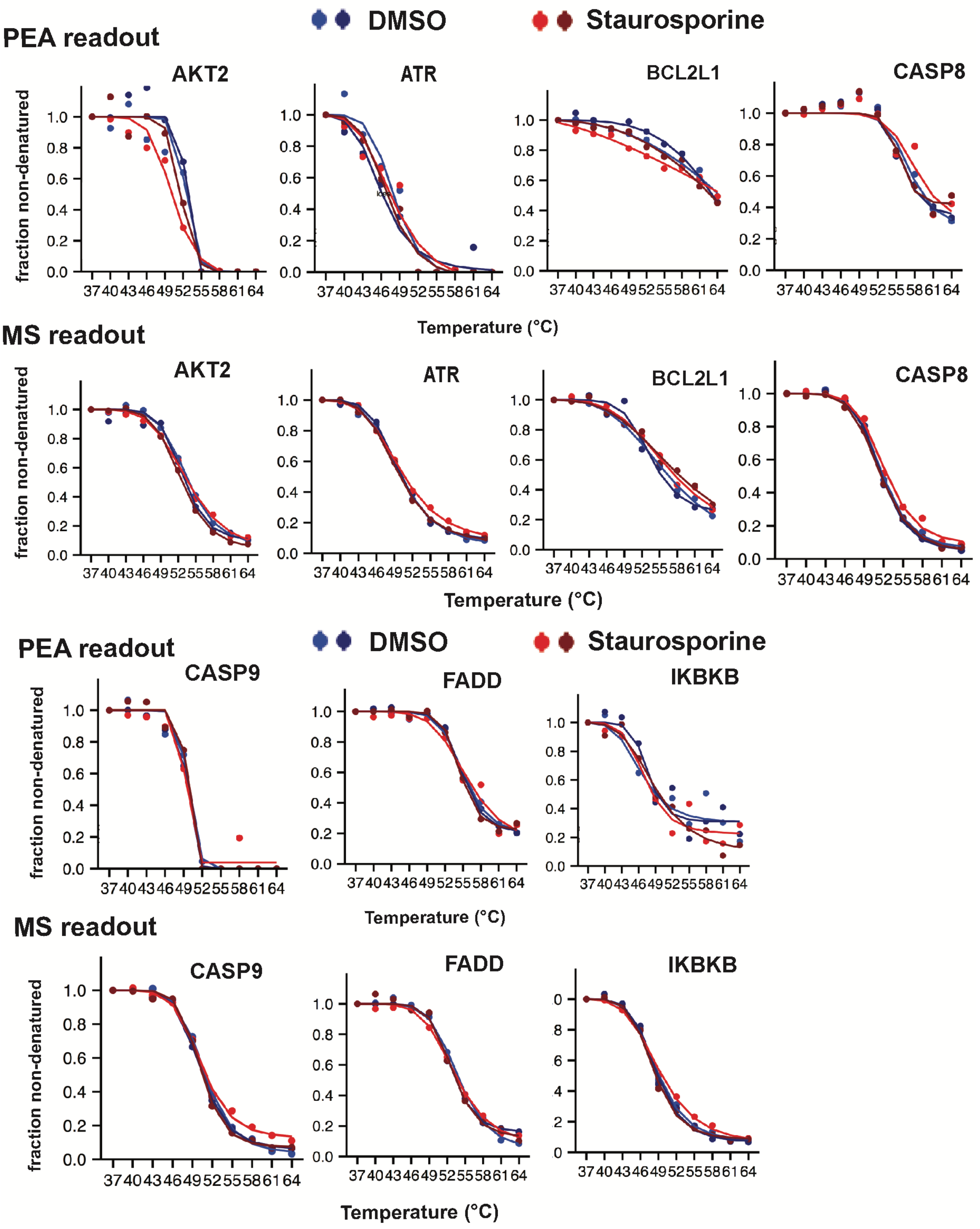

DMSO
Staurosporine
PEA readout
AKT2
ATR
BCL2L1
CASP8
fraction non-denatured
Temperature (°C)
MS readout
AKT2
ATR
BCL2L1
CASP8
fraction non-denatured
Temperature (°C)
PEA readout
DMSO
Staurosporine
CASP9
FADD
IKBKB
fraction non-denatured
Temperature (°C)
MS readout
CASP9
FADD
IKBKB
fraction non-denatured
Temperature (°C)

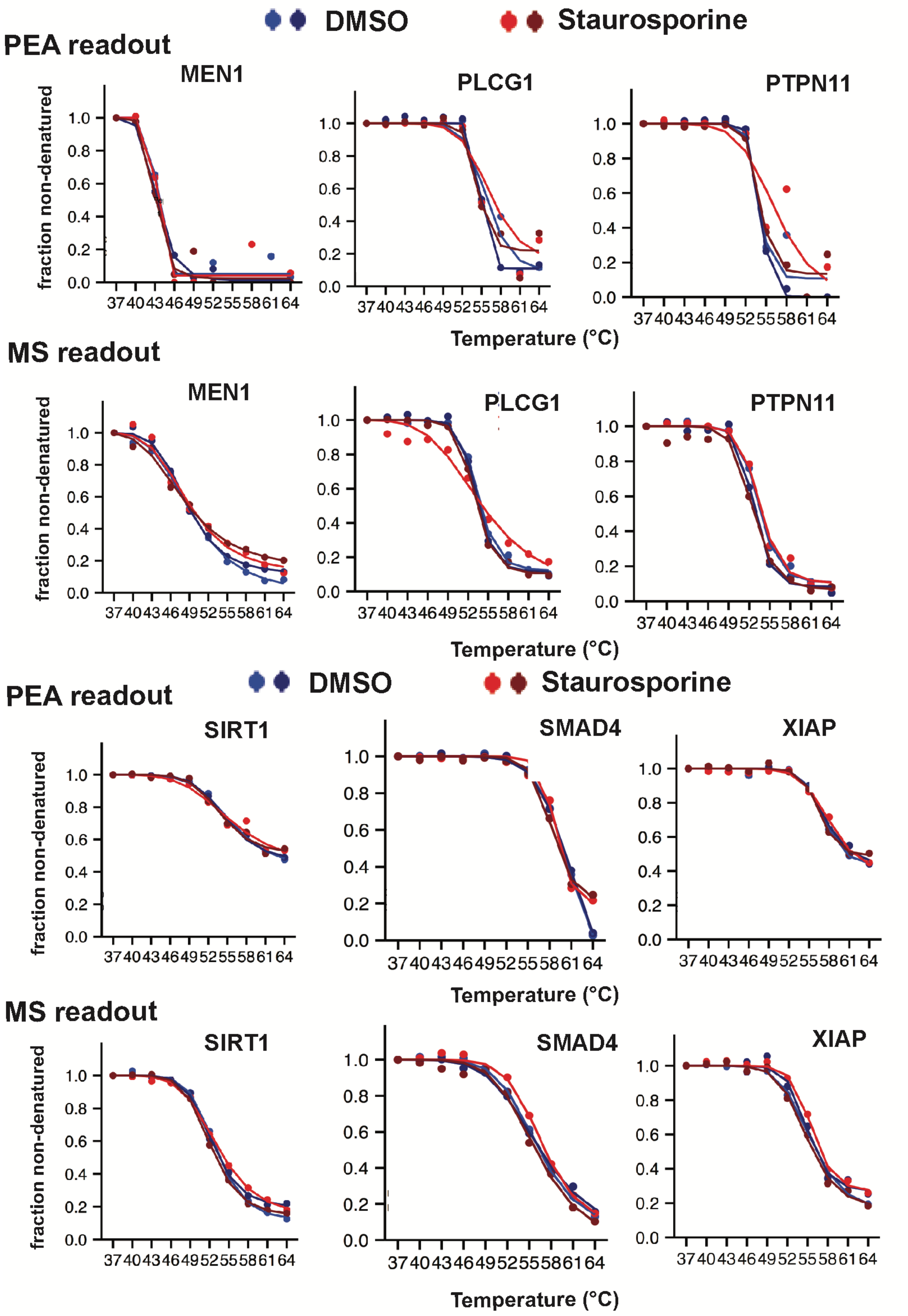

Figure S4. Examples of CETSA melting curves for proteins that were unaffected by staurosporine treatment. PEA and MS results exhibited good correlation for the proteins AKT2, ATR, BCL2L1, CAS8, CASP9, FADD, IKBKB, MEN1, PLCG1, PTPN11, SIRT1, SMAD4, TAKS and XIAP.

Figure S5.

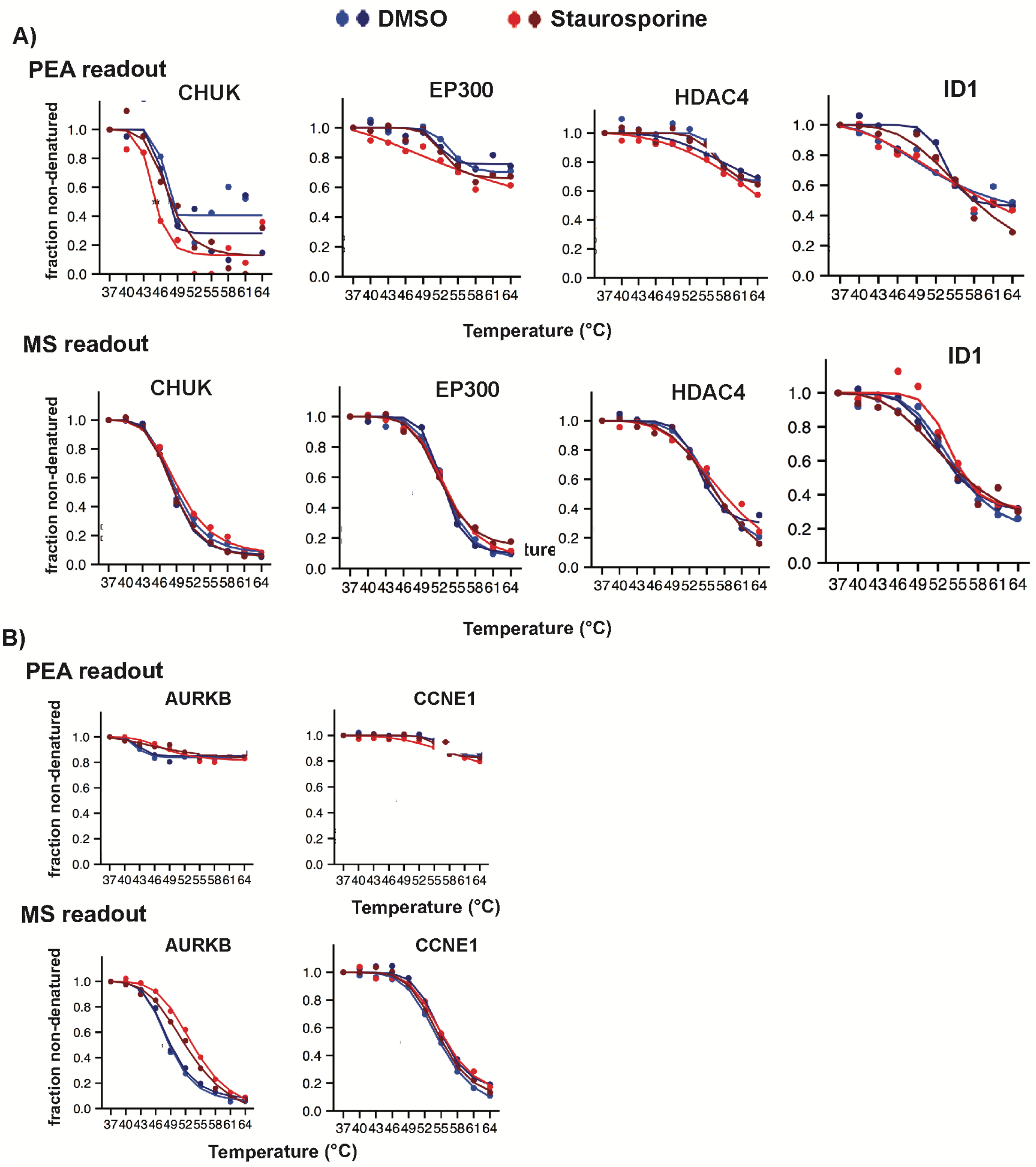

Figure S5. Examples of CETSA melting curves where PEA and MS readouts results exhibited some degree of discrepancy. (A) Moderate correlation between PEA and MS result for the proteins CHUK, EP300, HDAC4 and ID1. (B) Results for known target kinase for staurosporine AURKB and non-target kinase CCNE1 proteins measurement with poor correlation between PEA and MS melting curves results.

Figure S6.

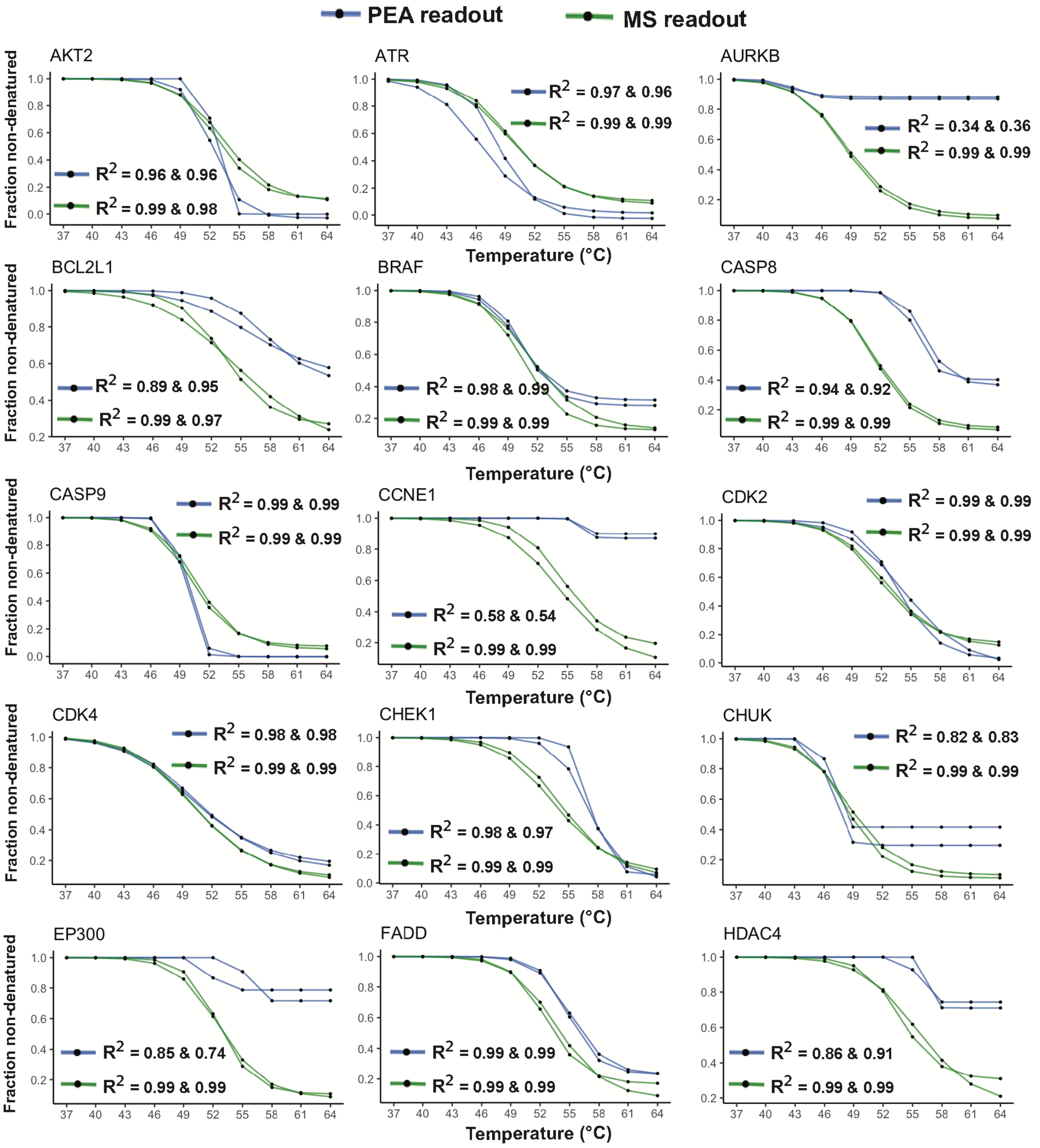
PEA readout
MS readout
Fraction non-denatured
Temperature (°C)
AKT2
R2 = 0.96 & 0.96
R2 = 0.99 & 0.98
ATR
R2 = 0.97 & 0.96
R2 = 0.99 & 0.99
AURKB
R2 = 0.34 & 0.36
R2 = 0.99 & 0.99
BCL2L1
R2 = 0.89 & 0.95
R2 = 0.99 & 0.97
BRAF
R2 = 0.98 & 0.99
R2 = 0.99 & 0.99
CASP8
R2 = 0.94 & 0.92
R2 = 0.99 & 0.99
CASP9
R2 = 0.99 & 0.99
R2 = 0.99 & 0.99
CCNE1
R2 = 0.58 & 0.54
R2 = 0.99 & 0.99
CDK2
R2 = 0.99 & 0.99
R2 = 0.99 & 0.99
CDK4
R2 = 0.98 & 0.98
R2 = 0.99 & 0.99
CHEK1
R2 = 0.98 & 0.97
R2 = 0.99 & 0.99
CHUK
R2 = 0.82 & 0.83
R2 = 0.99 & 0.99
EP300
R2 = 0.85 & 0.74
R2 = 0.99 & 0.99
FADD
R2 = 0.99 & 0.99
R2 = 0.99 & 0.99
HDAC4
R2 = 0.86 & 0.91
R2 = 0.99 & 0.99

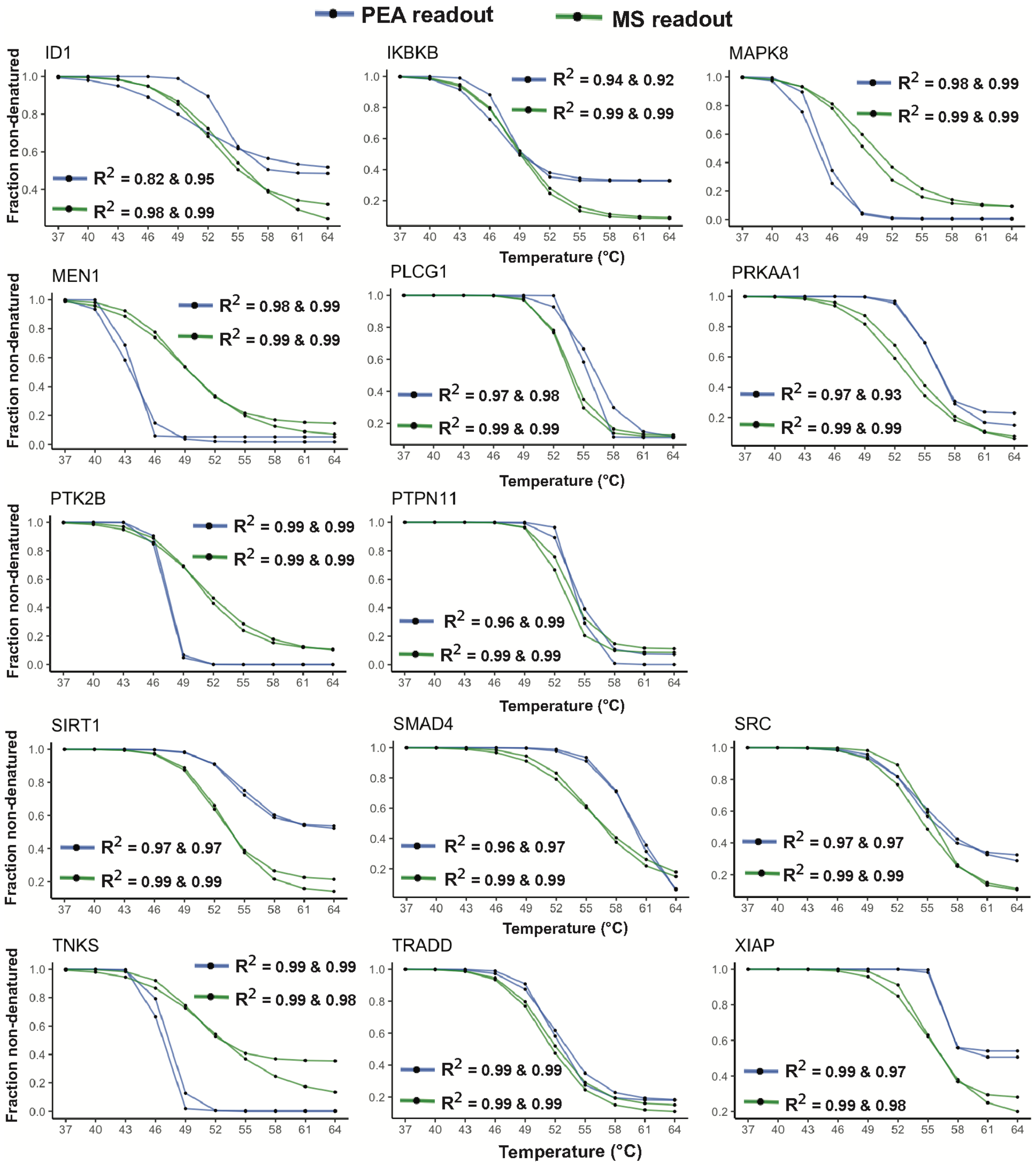

Figure S6. Quantitative correlation of PEA and MS readout of duplicate untreated samples for CETSA. The correlation is illustrated by results for 29 proteins in samples that were not treated with any drug. The data were normalized against the median values of total soluble protein levels for each detection method. For each analysis two biological replicates data were plotted for PEA (green) and MS (blue).

Figure S7.

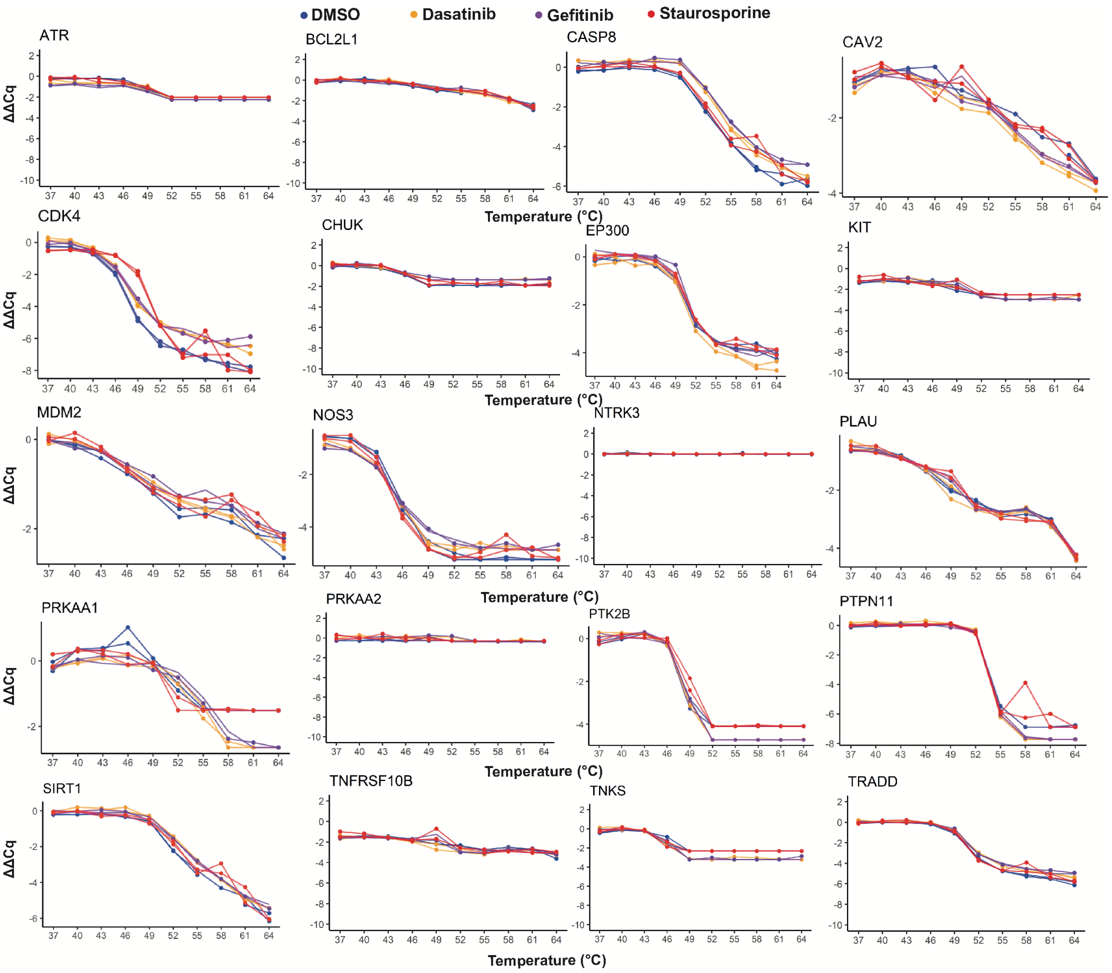

Figure S7. CETSA-PEA was used to study the effects by 20 µM of dasatinib (orange), gefitinib (purple) and staurosporine (red), or by the vehicle control DMSO (blue) for a set of proteins in K-562 cells. The figure illustrates CETSA-PEA melt curves for the known kinase targets CDK4, KIT, PRKAA1, PTK2B and TNKS for dasatinib, gefitinib and staurosporine, demonstrating variable compound-induced thermal shifts. PEA failed to detect the protein kinases NTRK3 and PRKAA2, all of which have very low to undetectable RNA expression in the investigated K-562 cells as reported in the Human Protein Atlas (www.proteinatlas.org). The proteins ATR, BCL2L1, CAS8, CHUK, EP300, MDM2, NOS3, PLAU, PTPN11, SIRT1, TNFRSF10B and TADD are known not to represent target proteins for the investigated compounds, and there was no clear evidence of compound-induced stabilization or destabilization.

References

(1) Assarsson, E.; Lundberg, M.; Holmquist, G.; Björkesten, J.; Thorsen, S. B.; Ekman, D.; Eriksson, A.; Rennel Dickens, E.; Ohlsson, S.; Edfeldt, G.; Andersson, A. C.; Lindstedt, P.; Stenvang, J.; Gullberg, M.; Fredriksson, S. Homogenous 96-plex PEA immunoassay exhibiting high sensitivity, specificity, and excellent scalability. *PloS One* **2014,** *9,* e95192.

(2) Lundberg, M.; Eriksson, A.; Tran, B.; Assarsson, E.; Fredriksson, S. Homogeneous antibody-based proximity extension assays provide sensitive and specific detection of low-abundant proteins in human blood. *Nucleic Acids Res.* **2011,** *39,* e102.

(3) Franzén, B.; Kamali-Moghaddam, M.; Alexeyenko, A.; Hatschek, T.; Becker, S.; Wik, L.; Kierkegaard, J.; Eriksson, A.; Muppani, N. R.; Auer, G.; Landegren, U.; Lewensohn, R. A fine-needle aspiration-based protein signature discriminates benign from malignant breast lesions. *Mol. Oncol.* **2018,** *12(9),* 1415–1428.

(4) Darmanis, S.; Gallant, C. J.; Marinescu, V. D.; Niklasson, M.; Segerman, A.; Flamourakis, G.; Fredriksson, S.; Assarsson, E.; Lundberg, M.; Nelander, S.; Westermark, B.; Landegren, U. Simultaneous multiplexed measurement of RNA and proteins in single cells. *Cell Rep.* **2016,** *14,* 380-389.

(5) Dziekan, J. M.; Wirjanata, G.; Dai, L.; Go, K. D.; Yu, H.; Lim, Y. T.; Chen, L.; Wang, L. C.; Puspita, B.; Prabhu, N.; Sobota, R. M.; Nordlund, P.; Bozdech, Z. Cellular thermal shift assay for the identification of drug-target interactions in the Plasmodium falciparum proteome. *Nat. Protoc.* **2020,** *15(6),* 1881–1921.

(6) Lim, Y. T.; Prabhu, N.; Dai, L.; Go, K. D.; Chen, D.; Sreekumar, L.; Egeblad, L.; Eriksson, S.; Chen, L.; Veerappan, S.; Teo, H. L.; Tan, C.; Lengqvist, J.; Larsson, A.; Sobota, R. M.; Nordlund, P. An efficient proteome-wide strategy for discovery and characterization of cellular nucleotide-protein interactions. *PloS One* **2018,** *13,* e0208273.

(7) Savitski, M. M.; Reinhard, F. B.; Franken, H.; Werner, T.; Savitski, M. F.; Eberhard, D.; Martinez Molina, D.; Jafari, R.; Dovega, R. B.; Klaeger, S.; Kuster, B.; Nordlund, P.; Bantscheff, M.; Drewes, G. Tracking cancer drugs in living cells by thermal profiling of the proteome. *Science* **2014,** *346,* 6205.

(8) Childs, D.; Bach, K.; Franken, H.; Anders, S.; Kurzawa, N.; Bantscheff, M.; Savitski, M. M.; Huber, W. Nonparametric analysis of thermal proteome profiles reveals novel drug-binding proteins. *Mol. Cell Proteomics* **2019,** *18,* 2506–2515.

(9) Benjamini, Y.; Hochberg, Y. Controlling the false discovery rate: a practical and powerful approach to multiple testing. *J. Roy. Statistical Soc.* **1995,** *57,* 289–300.

(10) Klaeger, S.; Heinzlmeir, S.; Wilhelm, M.; Polzer, H.; Vick, B.; Koenig, P. A.; Reinecke, M.; Ruprecht, B.; Petzoldt, S.; Meng, C.; Zecha, J.; Reiter, K.; Qiao, H.; Helm, D.; Koch, H.; Schoof, M.; Canevari, G.; Casale, E.; Depaolini, S. R.; Feuchtinger, A.; Wu Z.; Schmidt T.; Rueckert, L.; Becker, W.; Huenges, J.; Garz, A.K.; Gohlke, B.O.; Zolg, D.P.; Kayser, G.; Vooder, T.; Preissner, R.; Hahne, H.; Tõnisson, N.; Kramer, K.; Götze, K.; Bassermann, F.; Schlegl, J.; Ehrlich, H.C.; Aiche, S.; Walch, A.; Greif, P.A.; Schneider, S.; Felder, E.R.; Ruland, J.; Médard, G.; Jeremias, I.; Spiekermann, K.; Kuster, B. The target landscape of clinical kinase drugs. *Science* **2017,** *358,* 4368.

(11) Davis, M. I.; Hunt, J. P.; Herrgard, S.; Ciceri, P.; Wodicka, L. M.; Pallares, G.; Hocker, M.; Treiber, D. K.; Zarrinkar, P. P. Comprehensive analysis of kinase inhibitor selectivity. *Nat. Biotechnol.* 2011, *29,* 1046-1051.

(12) Bantscheff, M.; Eberhard, D.; Abraham, Y.; Bastuck, S.; Boesche, M.; Hobson, S.; Mathieson, T.; Perrin, J.; Raida, M.; Rau, C.; Reader, V.; Sweetman, G.; Bauer, A.; Bouwmeester, T.; Hopf, C.; Kruse, U.; Neubauer, G.; Ramsden, N.; Rick, J.; Kuster, B.; …

Drewes, G. Quantitative chemical proteomics reveals mechanisms of action of clinical ABL kinase inhibitors. *Nat. Biotechnol.* **2007,** *25,* 1035–1044.

(13) Kitagawa, D.; Yokota, K.; Gouda, M.; Narumi, Y.; Ohmoto, H.; Nishiwaki, E.; Akita, K.; Kirii, Y. Activity-based kinase profiling of approved tyrosine kinase inhibitors. *Genes to cells* **2013,** *18,* 110–122.

(14) Anastassiadis, T.; Deacon, S. W.; Devarajan, K.; Ma, H.; Peterson, J. R. Comprehensive assay of kinase catalytic activity reveals features of kinase inhibitor selectivity. *Nat. Biotechnol.* **2011,** *29,* 1039–1045.

(15) Karaman, M. W.; Herrgard, S.; Treiber, D. K.; Gallant, P.; Atteridge, C. E.; Campbell, B. T.; Chan, K. W.; Ciceri, P.; Davis, M. I.; Edeen, P. T.; Faraoni, R.; Floyd, M.; Hunt, J. P.; Lockhart, D. J.; Milanov, Z. V.; Morrison, M. J.; Pallares, G.; Patel, H. K.; Pritchard, S.; Wodicka, L. M.; Zarrinkar, P. P. A quantitative analysis of kinase inhibitor selectivity. *Nat. Biotechnol.* **2008,** *26,* 127–132.

(16) Elkins, J. M.; Fedele, V.; Szklarz, M.; Abdul Azeez, K. R.; Salah, E.; Mikolajczyk, J.; Romanov, S.; Sepetov, N.; Huang, X. P.; Roth, B. L.; Al Haj Zen, A.; Fourches, D.; Muratov, E.; Tropsha, A.; Morris, J.; Teicher, B. A.; Kunkel, M.; Polley, E.; Lackey, K. E.; Atkinson, F. L.; Overington, J.P.; Bamborough, P.; Müller, S.; Price, D.J.; Willson, T.M.; Drewry, D.H.; Knapp, S.; Zuercher, W. J. Comprehensive characterization of the Published Kinase Inhibitor Set. *Nat. Biotechnol.* **2016,** *34,* 95–103.

(17) Vasta, J. D.; Corona, C. R.; Wilkinson, J.; Zimprich, C. A.; Hartnett, J. R.; Ingold, M. R.; Zimmerman, K.; Machleidt, T.; Kirkland, T. A.; Huwiler, K. G.; Ohana, R. F.; Slater, M.; Otto, P.; Cong, M.; Wells, C. I.; Berger, B. T.; Hanke, T.; Glas, C.; Ding, K.; Drewry, D. H.; … Robers, M. B. Quantitative, wide-spectrum kinase profiling in live cells for assessing the effect of cellular ATP on target engagement. *Cell Chem. Biol.* **2018,** *25,* 206–214.

(18) Fabian, M. A.; Biggs, W. H.; 3rd, Treiber, D. K.; Atteridge, C. E.; Azimioara, M. D.; Benedetti, M. G.; Carter, T. A.; Ciceri, P.; Edeen, P. T.; Floyd, M.; Ford, J. M.; Galvin, M.; Gerlach, J. L.; Grotzfeld, R. M.; Herrgard, S.; Insko, D. E.; Insko, M. A.; Lai, A. G.; Lélias, J. M.; Mehta, S. A.; Milanov, Z.V.; Velasco, A.M.; Wodicka, L.M.; Patel, H.K.; Zarrinkar, P.P.; Lockhart, D. J. A small molecule-kinase interaction map for clinical kinase inhibitors. *Nat. Biotechnol.* **2005,** *23,* 329–336.

(19) Martinez Molina, D.; Jafari, R.; Ignatushchenko, M.; Seki, T.; Larsson, E. A.; Dan, C.; Sreekumar, L.; Cao, Y.; Nordlund, P. Monitoring drug target engagement in cells and tissues using the cellular thermal shift assay. *Science* **2013,** *341,* 84–87.

(20) Martinez Molina, D.; Nordlund, P. The cellular thermal shift assay: A novel biophysical assay for in situ drug target engagement and mechanistic biomarker studies. *Annu. Rev. Pharmacol. Toxicol*. **2016,** *56,* 141–161.

(21) Dai, L.; Prabhu, N.; Yu, L. Y.; Bacanu, S.; Ramos, A. D.; Nordlund, P. Horizontal cell biology: Monitoring global changes of protein interaction states with the proteome-wide cellular thermal ahift assay (CETSA). *Annu. Rev. Biochem.* **2019,** *88,* 383–408.

(22) Franken, H.; Mathieson, T.; Childs, D.; Sweetman, G. M.; Werner, T.; Tögel, I.; Doce, C.; Gade, S.; Bantscheff, M.; Drewes, G.; Reinhard, F. B.; Huber, W.; Savitski, M. M. Thermal proteome profiling for unbiased identification of direct and indirect drug targets using multiplexed quantitative mass spectrometry. *Nat. Protoc.* **2015,** *10,* 1567−1593.
